# Unmated Queens Show Worker‐Like Behaviour and Gene Expression in Polygynous Colonies of the Ant *Stigmatomma pallipes*

**DOI:** 10.1111/mec.70360

**Published:** 2026-04-29

**Authors:** Maximilian F. Bolder, Jannik S. Möllmann, Thomas J. Colgan, Romain Libbrecht

**Affiliations:** ^1^ Institute of Organismic and Molecular Evolution, Johannes Gutenberg University Mainz Germany; ^2^ Insect Biology Research Institute, UMR 7261, CNRS, University of Tours Tours France

**Keywords:** Amblyoponinae, division of labor, phenotypic plasticity, social insects, transcriptomics

## Abstract

Insect societies show a reproductive division of labor between egg‐laying queens and workers that fulfil all non‐reproductive tasks. Polygyny, the coexistence of several queens in a colony, has evolved multiple times in social insects. Although queens in polygynous colonies are often assumed to have similar reproductive outputs, they may actually show variation in terms of physiology and behaviour. However, little is known on the mechanistic basis of such variation. Here, we used the ant *Stigmatomma pallipes* to investigate: (i) whether nestmate queens from polygynous colonies differed in mobility, behaviour, and gene expression; and (ii) whether this variation was explained by their mating status. We used individual tracking and behavioural scoring to identify two types of queens: low‐mobility (LM) queens that behaved like monogynous queens and high‐mobility (HM) queens with worker‐like behaviour. Dissections of reproductive organs revealed that only one queen per colony was mated and reproductively active, consistently corresponding to the LM category. As a complementary approach, we performed transcriptomic‐based analyses of brains and fat bodies, finding that mated LM‐polygynous queens had similar transcriptomic profiles to monogynous queens, while unmated HM‐polygynous queens resembled workers. Transcriptomic differences between LM‐ and HM‐polygynous queens were primarily associated with processes related to protein synthesis, transcription, and neural activity. Our finding that unmated queens in polygynous *S. pallipes* colonies exhibit worker‐like behaviour and gene expression challenges traditional views of queen specialization at different biological levels and provides new insights into reproductive division of labor in insect societies.

## 1 Introduction

The reproductive division of labor is considered a key factor underlying the ecological success of social insects (Wilson 1978; Miura et al. 2022; Muratore et al. 2023). In these societies, a morphologically and behaviourally specialized queen caste monopolizes egg production (William Morton Wheeler 1911; Boomsma and Gawne 2018; Hellemans and Hanus 2024) and has often been compared to the highly specialized germline of a multicellular organism, dedicated solely to reproduction (William Morton Wheeler 1911; Szathmáry and Smith 1995; Boomsma and Gawne 2018; Miura et al. 2022; Majidifar et al. 2024). Nevertheless, queens can express a broad range of behaviours, both during colony foundation as well as in established colonies (Lenoir and Dejean 1994; Brown and Bonhoeffer 2003; Evans and Raine 2014; Ortiz‐Alvarado and Rivera‐Marchand 2020; Mizumoto et al. 2021; Majidifar et al. 2024). In species where multiple queens coexist in a colony (polygyny), this behavioural repertoire may diversify further, as reproductive and social roles may be distributed among queens.

In the ants, polygyny occurs frequently and can take multiple forms, which are not limited to the presence of multiple queens with equal reproductive outputs and similar behaviour (Hölldobler and Wilson 1977; Keller 1991; Gadau and Fewell 2009; Boomsma et al. 2014). These differences were observed in colonies with dominance hierarchies (Ito et al. 1996) or with coexisting mated and unmated dealate (wingless) queens (Buschinger 1968). Subordinate and/or unmated queens typically behave like non‐reproductive workers, performing actions such as foraging, defense, and brood care (Petersen‐Braun 1975; Ito et al. 1996; Peeters 1997; Hora et al. 2005; Nehring et al. 2012; Vieira et al. 2012; Araújo et al. 2016; Murakami 2020). These cases illustrate that the queen caste can encompass multiple behavioural and physiological profiles; nevertheless, the molecular background of this variation and how it compares to the worker caste remain poorly understood.

There is no evidence that the observed variation among queens stems from genetic differences, but rather from phenotypic plasticity, whereby individuals with nearly identical genetic backgrounds develop distinct morphological and behavioural profiles due to differences in gene expression (Grozinger et al. 2007; Bonasio et al. 2010; Taylor et al. 2021; Xu and Colgan 2025). Phenotypic plasticity not only produces the morphologically differentiated queen and worker castes (Wilson 1953; Wheeler 1986; Gadagkar 1997; Berens et al. 2015; Corona et al. 2016) but also behavioural variation within the worker caste (Wilson 1953; Wheeler 1986; Toth and Robinson 2007; Smith et al. 2008; Mikheyev and Linksvayer 2015). Such worker behavioural plasticity at the adult stage is associated with distinct transcriptomic profiles, often in specific tissues, such as the brain or fat body (Corona et al. 2013, 2016; Nie et al. 2018; Libbrecht et al. 2018; Kohlmeier et al. 2019; Miyazaki et al. 2021; Qiu et al. 2022; Caminer et al. 2023). Previous transcriptomic studies on the queen caste have provided biological insights into how the molecular phenotype changes after mating (von Wyschetzki et al. 2015; Nagel et al. 2020; Liu et al. 2023), as well as changes associated with age (von Wyschetzki et al. 2015; Lucas et al. 2017; Lucas and Keller 2018; Nagel et al. 2020; Korb et al. 2021). To identify the molecular basis of behavioural variation among queens in polygynous colonies requires investigating the associated transcriptomic variation in relevant tissues. In this regard, the brain reflects differences in behavioural regulation, whereas the fat body provides complementary insights into metabolic and reproductive physiology.

To further our understanding of the functional repertoire of the queen caste in polygynous species, we investigated behavioural and transcriptomic variation within the queen caste in polygynous colonies of the ant *Stigmatomma pallipes*. This species is an exemplar system to study variation among queens because it is socially polymorphic, in that colonies can be monogynous or polygynous, with its polygynous colonies documented to contain queens that express worker‐like behaviours (William Morton Wheeler 1900; Haskins 1928; Haskins and Enzmann 1937; Traniello 1982), yet the bases and consequences for such variation are unknown. In addition, *S. pallipes* belongs to the Amblyoponinae, an understudied subfamily of ants with limited morphological differences between queens and workers. Using a mobility‐based approach to categorize queens, we investigated how queen types differ in mating status, behaviour, and transcriptomic profiles. This allowed us to characterize variation among queens across biological scales, ranging from the molecular to the organismal. To facilitate these investigations, we also generated one of the first genome assemblies for this subfamily. Together, these data provide a comprehensive overview of variation within queens in polygynous colonies, contributing to our understanding of the evolution and functional repertoire of the queen caste in social insects.

## 2 Methods

### 2.1 Colony Collection and Maintenance

Colonies of *S. pallipes* were collected under flat rocks at the Huyck Preserve, New York (42°30′56.23″N, 74°08′21.34″W) during May 2024. Entire colonies, including queens, workers, eggs, larvae, and pupae, were collected and transferred into plastic bags containing some of the surrounding soil and moist cotton. Upon arrival at the Johannes Gutenberg University of Mainz, Germany, the colonies were moved into plaster nests (14 cm Width × 16 cm Length × 6 cm Height). The nests featured a central depression covered with a glass slip to allow congregation, and the nest was subsequently covered with soil, which was kept moist. Colonies were maintained in a temperature‐controlled room at 22°C in the dark, as *S. pallipes* workers do not have functional eyes, and were fed twice weekly with chopped mealworms.

### 2.2 Experimental Manipulations and Video Recording

For our study, we used 25 polygynous (ranging between two to six queens) and five monogynous colonies of *S. pallipes* (Table S1). To examine mobility variation among queens, the soil and glass slip, under which the ants nested, were removed, and queens were individually marked with dye (blue, green, orange, pink, purple, red, white, and yellow) from a non‐toxic marker pen (Edding 750, Edding, Germany) on the thorax and abdomen. In cases where queens lost the marking or were missed during marking, they were assigned the unmarked tag (no colony had more than one unmarked queen). After a one‐week acclimation period to allow ants to resettle after the marking, the overlying soil was removed from each nest and remained removed for the duration of the experiment. The 30 colonies were divided into two batches of 15 colonies, which were filmed 24 h apart. Over two weeks, each colony was filmed five times, with each filming (hereafter, called “observation”) lasting 110 min. During each observation, a colony was filmed under an individual camera (Sony FDR‐AX33 Handycam, Sony Corporation, Japan) equipped with a ring light (Selfie Ring Light WLD‐S1, RealPlus, China). The camera's field of view encompassed the entire nest and immediate surrounding area. To account for possible positional and time‐of‐day effects, we randomized the order of filming and camera assignments. After the fifth and final observation, the entire colony of each nest was extracted immediately and separated into multiple cryotubes. Cryotubes were flash‐frozen using liquid nitrogen and transferred to a −80°C freezer for storage prior to downstream analyses.

### 2.3 Mobility Measurement

To test individual differences in queen mobility, the distance travelled by queens between frames was calculated by tracking their positions over time. To do so, we extracted, for each observation, the first frame of every minute using a custom script in FFmpeg v.4.2.7. (FFmpeg developers 2024), omitting the first and last 10 min of each video to avoid time points where disturbances could have occurred. This resulted in 91 frames per observation. In each frame, we marked the thorax position of each queen manually using a custom macro script in ImageJ v.1.54f (Schindelin et al. 2012) to load in images and save positions. In the first frame of each observation, we also marked the nest entrance and all four nest corners. Pixel distances were transformed into millimetres using a scale factor specific to each recording. This factor was based on the known length (12 cm) or height (8 cm) of the glass slip divided by the respective measured pixel distance. When queens were not visible due to exiting the nest, their location was marked at a single point outside the nest boundary. This occurred in 1166 out of 40,039 frames (2.9%). For consecutive unobservable points, we estimated the distance travelled using the global mean stepwise distance across all queens and observations (5.27 mm).

To investigate whether polygynous queens differed in mobility, we used the R package lme4 v.1.1‐36 (Bates et al. 2003) to build for each polygynous colony a linear mixed‐effects model with the distance travelled between frames as response variable, the queen identity as explanatory variable and the observation as random effect. We ran ANOVAs using the package car v.3.1.3, (Fox et al. 2001) to test for each colony whether there was a main effect of queen identity. From each of the 25 models, we extracted the estimated marginal mean of the distance travelled for each queen (hereafter, referred to as “mobility”) using the emmeans package v.1.11.1 (Lenth 2025). In monogynous colonies, we calculated the mean distance travelled by the queen across all observations to estimate mobility. We used the highest mobility value of monogynous queens, rounded to the nearest whole number (4 mm), as a threshold to classify queens in polygynous colonies. Queens with a mobility value above this threshold were classified as high‐mobility (HM‐polygynous queens), and those with a mobility value below the threshold as low‐mobility (LM‐polygynous queens).

To further investigate how the differences in mobility were associated with behaviour, mating status, and gene expression, we selected the polygynous colonies for which we found evidence of mobility variation among queens and that contained at least one HM‐ and one LM‐polygynous queen. Fifteen of the 25 polygynous colonies met these criteria. Ten colonies did not meet the criteria because all queens were above the threshold (six colonies), all queens were below the threshold (three colonies), or there was no evidence of mobility variation among queens (one colony). We also used the queens from the five monogynous colonies. From each polygynous colony, one HM‐ and one LM‐queen were chosen. In the case that multiple queens were on either side of the designated threshold, one queen was selected at random. In total, 30 queens from polygynous colonies and five queens from monogynous colonies were used for further analyses. Additionally, we added a single worker from each colony as an outgroup for the mating status and gene expression analyses. This resulted in a total of 55 specimens: 15 LM‐polygynous queens; 15 HM‐polygynous queens; five monogynous queens; and 20 workers.

### 2.4 Tissue Sampling for Gene Expression Analyses

To understand how queens differed in terms of gene expression, we focused on two tissues: the brain, which is important for behaviour and locomotion, and the fat body, which plays key roles in metabolism and reproduction. Dissections were performed in a randomized order and were blind to mobility status. The brain and fat body of each individual were dissected on separate dates.

To dissect the brain, the head was removed and transferred to a drop of 1× PBS solution on an ice‐cooled silicone slide. The rest of the body was returned to the cryotube and kept in liquid nitrogen. During each of the following steps, one microforceps (No. 11295‐10, F.S.T, Canada) was used to hold the head in place by grabbing a piece of cuticle or the mandibles. The antennae were removed at the base. The head was opened by cutting dorsally into the head capsule with a microscissors (No. 15000‐08, F.S.T., Canada), starting from the clypeus and moving up to the ocelli and the vertex along the frontal line. This was followed by two cuts at 90° to the first, in the direction of the eyes, resulting in a cross‐like cut. The cuticle encompassing the cut was then removed using two microforceps. Once the cuticle towards the eyes had been removed, one microforceps was used to rub the inside of the compound eyes on both sides of the head, severing the connection to the retina. This step was skipped for workers, as they lack well‐developed eyes. The rest of the cuticle obscuring the brain was removed and the brain was carefully extracted by grabbing it from below at the posterior end and lifting it out of the head capsule and into another cooled 1× PBS droplet. It was then transferred to a pre‐chilled vial containing 100 μL of TRIzol and incubated on ice. After dissection, all samples were transferred and stored at −80°C.

To sample the fat body, the gaster was dissected. The gaster was separated at the petiole from the thorax, and cut into two parts by making an incision with the microscissors between the second and third abdominal segments, while holding it in place with a microforceps. The lower half of the gaster was removed and placed into a separate 1× PBS droplet for later assessments of mating status. From the upper half, which comprised the first and second abdominal segments, we removed the remaining internal trachea, gastrointestinal tract, and components of the nervous system. In addition, the ventral parts of the second abdominal segments were removed until only parts of it and the first abdominal segments with subcuticular fat body remained. Fat body with remnants of the first and second abdominal segments was transferred into a vial with 100 μL TRIzol and stored at −80°C.

### 2.5 Assessment of Mating Status

The mating status and ovarian development of all queens from colonies with significantly different mobility were assessed, as well as those of the monogynous queens and a single worker from each colony. To reveal the ovaries, we dissected the lower half of the gaster in ice cooled 1× PBS, removing the remaining cuticle, the gastrointestinal tract, and the aculeus (stinger). The ovaries were transferred to a glass slide and imaged with the built‐in camera of the microscope (Leica S9i, Leica Microsystems, Germany). To measure ovarian development, ovaries with white ovarioles, often accompanied by yellow bodies, were scored as developed. Ovaries lacking these features were scored as undeveloped. To assess the mating status, we scored the presence of a visible spermatheca, as well as whether it was filled or empty. Individuals with a filled spermatheca were classified as mated, while individuals with either an empty spermatheca or no visible spermatheca were scored as unmated. The scoring of ovarian development and mating status was performed blind to mobility status. We conducted Fisher's exact tests to investigate the association between mobility and mating status, and between mobility and ovarian development.

### 2.6 Behavioural Analyses

To investigate whether queens with different mobility express different behavioural patterns, we used the BORIS software v.7.13.9 (Friard and Gamba 2016) to score a total of 13 behaviours (Table 1).

**TABLE 1:** Summary table of all queen behaviours that were scored in the behavioural analysis.

Behaviour	Description
Resting	Not moving legs/no observable movements
Walking	Moving around
Digging	Removing soil or plaster
Nest exit	Exiting the nest, not being visible to the observer
Queen‐queen interaction	Interaction between queens, such as grooming, aggressive behaviour, intense antennation
Queen‐worker interaction	Interaction between queens and workers such as grooming, aggressive behaviour, intense antennation
Brood care	Actively licking or manipulating the brood (antennation alone was not considered as brood care)
Brood carrying	Carrying brood around
Carrying other	Carrying soil or food items around
Feeding on food items	Feeding on food items such as pieces of mealworms
Hemolymph feeding	Squeezing larvae to feed on their hemolymph
Self‐grooming	Cleaning and licking own legs, antennae, mandibles and/or gaster
Egg laying	Flexing the gaster and visibly producing an egg

We scored the behaviour of each selected queen during the first 10 s of every minute across each of the five 90 min observations, for a total of 91 scans per observation and 455 behavioural scans per individual. Each scan was associated with a single behaviour. If multiple behaviours were observed during one scan, the behaviour performed the longest was scored.

To analyse behavioural variation among the three queen categories (monogynous queens, LM‐polygynous queens, HM‐polygynous queens), we first pooled for each queen all scans from the five observations after verifying that queen behaviour was consistent across observations by visual inspection (Figure S1). We then analysed behavioural differences among queens by performing a principal component analysis (PCA) on scaled and centered summarized behavioural counts across all observations. We conducted *K*‐means clustering using all principal components (PCs) to identify clusters of queens that shared similar behavioural profiles. Cluster assignments were visualized on the PCA, and their association with mobility categories of the queens (monogynous, LM‐ and HM‐polygynous queens) was evaluated using Fisher's exact tests.

We further analysed the eight PCs that were needed to account for 90% of the total variance. We tested the main effect of queen type on PC scores by conducting ANOVAs on the output of linear mixed‐effects models that included colony as a random factor. Post hoc pairwise tests were performed using the emmeans package v.1.11.1 (Lenth 2025) with Tukey HSD adjustment for multiple comparisons. We identified the behaviours that were primarily associated with each PC as those with loading values exceeding 75% of the maximum loading value.

### 2.7 RNA Extraction, Library Preparation, and Sequencing

To investigate transcriptomic variation, we used the aforementioned 55 individuals (15 LM‐polygynous queens, 15 HM‐polygynous queens, five monogynous queens, and 20 workers). From each individual, we extracted total RNA from the brain and fat body separately using a trizol:chloroform extraction protocol followed by purification using the Qiagen RNeasy extraction kit. This resulted in a total of 110 RNA samples that were shipped on dry ice to a commercial supplier (Novogene, Germany) that conducted quality assessment using an Agilent Bioanalyzer and individual mRNA‐enriched library preparations using the NovogeneNGS RNA Library Prep Set (PT042) property kit. The libraries were individually barcoded, pooled, and sequenced on an Illumina Plus X sequencing platform. Sequencing failed for eight samples: six from the brain (two LM‐polygynous queens, two HM‐polygynous queens, and two workers); and two from the fat body (one LM‐polygynous queen and one worker) (Table S2), reducing our dataset to 102 samples. For these samples, the amount of sequencing data ranged from 8 to 76 million paired‐end (2*150 bp) reads per sample (Table S2).

### 2.8 Sample Collection and DNA Extraction for Genome Assembly

To allow for read alignment and associated transcript quantification, a *de novo* genome assembly was generated as a genome assembly for *S. pallipes* was not available at the onset of our analysis. To provide sufficient input material for DNA sequencing, we collected a total of 24 pupae from a single colony that was not part of the behavioural experiment. For the following steps, the 24 pupae were subsampled into sets of two, generating a total of 12 pupal sets. Each pupal set was homogenized in 460 μL of homogenization buffer with 40 μL of 10% Triton X‐100. The resulting homogenate was filtered through a 70 μm filter while kept on ice. The retained substrate was resuspended in 400 μL of homogenization buffer and homogenized again before being transferred to a 1.5 mL Eppendorf tube. An additional 500 μL of homogenization buffer was added, and the mixture was centrifuged at 1,000 rpm for 5 min at 4°C. The supernatant was discarded, and the pellet was resuspended in 500 μL of homogenization buffer. This process was repeated once more after subsequent centrifugation. The pellet was then resuspended in 200 μL of homogenization buffer, to which 5 μL of proteinase K, 20 μL of 10% sodium dodecyl sulfate (SDS), and 20 μL of dialysis buffer were added. The mixture was carefully inverted and incubated at 56°C and 600 rpm for 30 min on a heat block, followed by an additional 10 min incubation at the same temperature.

After incubation, the pupal sets were pooled. To this combined sample, a 0.1× volume of Tris and a 0.25× volume of sodium perchlorate (NaClO_4_) were added. This was followed by a phenol:chloroform:isoamyl extraction, including one re‐extraction of the lower phase with 500 μL homo‐buffer. This step was followed by a further chloroform: isoamyl alcohol extraction. The aqueous supernatant was collected and treated with an RNase at room temperature for 30 min. The DNA was precipitated by adding a 0.1× volume of 10× dialysis buffer and two volumes of ethanol. The samples were centrifuged at 15,000 rpm and the supernatant was discarded. The DNA pellet was dissolved in 30 μL HPLC‐grade water and incubated at 4°C for 12 h. For library preparation, we used Nanopore's native barcoding kit (SQK‐NBD114.24), according to the protocol provided by ONT with minor modifications, which included an extended incubation time for ligation and a bead clean up.

### 2.9 DNA Sequencing, Genome Assembly, and Annotation

Genomic libraries were sequenced on an Oxford Nanopore Technologies Promethion flow cell. After sequencing, raw pod5 files were basecalled using dorado v.0.7.0 + 71 cc744 using model ‘dna_r10.4.1_e8.2_400bps_sup@v5.0.0’. The initial number of raw sequences was 6,662,475 reads (mean size: 2,606 bp, maximum size: 675,316 bp). We performed quality checks of raw sequences using NanoPlot v.1.44.1 (de Coster and Rademakers 2023) with sequences then filtered using chopper v.0.10.0 (de Coster and Rademakers 2023) to remove sequences with mean base quality less than 10. Using these filtered reads, we next performed a *de novo* assembly using flye in high accuracy mode ‐nano‐hq, v.2.9.1 (Kolmogorov and Yuan 2019) with a minimum overlap length of 1 kb. Initial quality checks of the resulting genome assembly were performed using QUAST v.5.3.0 (Gurevich et al. 2013), which provides summary statistics on the assembly.

To correct base‐level errors and fix mismatches, genome polishing was performed using Racon v.1.5.0 (Vaser et al. 2017), which involved three rounds of polishing and corrects for minor base errors. For each polishing round, reads were remapped against intermediate polished assemblies using minimap2 v.2.28 (Li 2018). This approach was complemented by using Medaka v.2.0.1 (https://github.com/nanoporetech/medaka), a neural network‐based polisher that further improves base‐level accuracy after initial base corrections. Using the medaka‐polished genome assembly, RepeatModeler v.2.0.2 (https://github.com/Dfam‐consortium/RepeatModeler) was next used to discover and identify putative repetitive regions in the genome assembly, which were subsequently softmasked using RepeatMasker (https://www.repeatmasker.org).

Lastly, the consensus polished assembly was assessed for coverage by backmapping initial filtered reads against the polished assembly using minimap2. Contigs less than 1,000 bp were removed. This final assembly, which consisted of 822 contigs (total length: 232.4 Mb), was assessed for completeness through comparisons with both the latest Insecta and Hymenoptera BUSCOs (Benchmarking Universal Single‐Copy Orthologs).

To annotate the genome assembly, we generated transcriptome‐informed gene model predictions. First, we performed data quality evaluations for all 102 samples. For each sample, the general quality metrics were generated and examined using FastQC v.0.12.1 (Andrews 2010) providing information on base quality and potential adapter contamination. As a complementary approach, the proportion of raw reads mapping to the generated genome assembly was investigated using HISAT v2.2.1 (Kim et al. 2019), finding high mapping rates (mean: 91.55%). The outputs were combined and visualized for both sets of quality assessments using MultiQC v.1.29 (Ewels et al. 2016). Based on the results of the quality assessment, adapter sequences were removed, which can affect mapping rates and quality, and reads filtered by quality (PHRED score ≥ 15) and length (minimum length ≥ 50 bp) using fastp v.0.20.1 (Chen et al. 2018). Due to low read count, two samples were removed prior to subsequent gene model prediction.

The filtered reads were then used in conjunction with a protein database comprised of arthropod‐derived proteins from OrthoDB v12 (Kuznetsov et al. 2023), published hymenopteran proteins (Kapheim et al. 2015), and all proteins from the curated Swiss‐Prot database (The UniProt Consortium 2023) to predict gene models within the softmasked genome assembly using BRAKER3 v3.0.8 (Gabriel et al. 2024). Initial predictions were further quality filtered and processed to identify and correct putative incorrect gene models. To this end, predicted genes with no overlap among their transcripts were split into separate genes, and gene predictions with missing start or stop codons, or premature stop codons were removed to retain only the highest quality predictions. As predicted gene models occasionally included very short introns of 1–3 bp, which are highly likely to represent technical errors, we joined adjacent exons if they were separated by less than 4 bp. Finally, quality‐filtered gene model predictions were then translated to predicted proteins and used as input to Compleasm v.0.2.7, mode: “protein” (Huang and Li 2023; Manni et al. 2021), resulting in a BUSCO completeness of 97.06% (database: hymenoptera_odb12, *n* = 5,912). This level of completeness is comparable to the assembly's BUSCO completeness of 97.89% (mode: “assembly”, database: hymenoptera_odb12, *n* = 5,912), indicating that most genes that were correctly assembled were also annotated with gene model predictions.

### 2.10 Differential Expression and GO Term Enrichment Analyses

For all RNA‐seq samples, raw paired‐end reads were filtered using fastp v.0.20.1 (Chen et al. 2018) to remove reads containing more than 15 ambiguous bases (“N”s) and reads shorter than 120 bp, with filtered reads saved separately for forward and reverse pairs. Afterwards, a quality assessment of filtered reads was performed via FastQC v.0.12.1 (Andrews 2010), with output files summarized and visualized using MultiQC v.1.29 (Ewels et al. 2016). Transcript abundances were next quantified per sample by pseudo‐aligning filtered RNA‐seq data against predicted coding regions informed from our *de novo*‐assembled genome using Kallisto v.0.50 (Bray et al. 2016). This resulted in transcript abundance estimates for all 102 sample (49 brain samples: five monogynous queens; 13 HM‐polygynous queens; 13 LM‐polygynous queens; and 18 workers; 53 fat body samples: five monogynous queens; 15 HM‐polygynous queens; 14 LM‐polygynous queens; and 19 workers), which were loaded into R using tximport v.1.37.2 (Soneson et al. 2016), summarized to gene‐level counts and analysed using the R package DESeq2 v.1.49.4 (Love et al. 2014). Prior to differential expression analysis, we filtered out genes with less than five reads in five samples.

To investigate transcriptomic variation among the four individual types, we used variance‐stabilized transformed counts to perform two separate PCAs: one for the brain samples; and one for the fat body samples. Next, we applied *K*‐means clustering to all PCs to identify clusters of samples that share relatively similar transcriptomic profiles. We subsetted polygynous queen samples to test the association between cluster membership and polygynous queen mobility status types using a Fisher's exact test and quantified the strength of the association with Cramer's V.

To assess whether gene expression differed among individual types, we used likelihood ratio tests (LRT) from DESeq2. We tested the main effect of individual type on gene expression by comparing a full model that included individual type to a reduced model that did not. Genes with a Benjamini‐Hochberg adjusted *p*‐value of less than 0.05 were considered differentially expressed genes (DEGs), as the analysis provided evidence that their expression differed between at least two of the four individual types. We clustered the DEGs using the DEGreport package v.1.42.0 (Pantano 2025), to produce gene clusters that contain DEGs with similar expression differences across individual types.

We investigated whether DEGs in gene clusters that encompassed more than 5% of all DEGs were enriched for biological processes using Gene Ontology (GO) term enrichment analyses with the topGO package v.2.52.0 (Adrian Alexa 2017). GO annotations for *Stigmatomma pallipes* genes were inferred through homology to *Drosophila melanogaster* using OrthoFinder v.2.5.4 (Emms and Kelly 2015). Predicted protein sequences, generated from our assembled and annotated *S. pallipes* genome, were used as input for our homology‐based inference analysis along with predicted proteins from the latest *D. melanogaster* genome assembly. The resulting homologous mappings were used to assign GO terms to *S. pallipes* genes based on annotations assigned to the homolog in the fruit fly. The GO annotations were imported as a gene‐to‐term mapping table in R and converted into a list structure compatible with topGO. In total, 7323 genes were annotated with a non‐redundant list of 8572 GO terms. Genes without GO annotations or not expressed in the dataset, as well as GO terms with less than five annotated genes (node size = 5), were excluded from our analysis, with the remaining annotated genes defined as the background gene universe.

For the enrichment analysis, a separate background gene universe was generated for each tissue, consisting of all expressed genes with a GO annotation (brain: 2951 genes; fat body: 1863 genes). These numbers represented the subset of the 7323 annotated genes that were expressed in each respective tissue. Of these, 2736 and 1715 genes, respectively, could be mapped to GO terms in the Directed Acyclic Graphs (DAGs) used by topGO. The GO DAGs used contained 5794 terms with 12,206 relations (i.e., directional links between GO terms indicating hierarchical relationships) for the brain and 2771 terms with 10,592 relations for the fat body. A binary gene list indicating the presence (1) or absence (0) of genes in each cluster, relative to the full gene universe, was created. Functional enrichment was tested separately for each cluster using the “weight01” algorithm, and Fisher's exact tests applied to terms from the Biological Process (BP) ontology. Resulting *p*‐values were adjusted for multiple testing using the Benjamini–Hochberg correction (via p.adjust() in R) before applying a significance threshold of adjusted *p* < 0.05. Enrichment results were retrieved using the GenTable function and GO term descriptions were appended using the GO.db R package v.3.15.0 (Carlson, 2017).

## 3 Results

### 3.1 Queens Differed in Mobility in Polygynous Colonies

**FIGURE 1:**
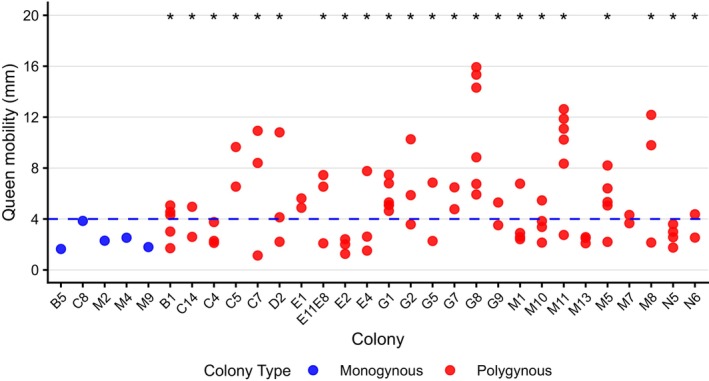
Mobility of *Stigmatomma pallipes* queens differed in polygynous colonies, with some having higher values and some similar values to monogynous queens. A dot plot highlighting evidence for variation among queens in mobility in most polygynous colonies (marked with an asterisk). Many polygynous queens (red dots) expressed higher mobility than monogynous queens (blue dots). The dashed blue line at 4 mm indicates the threshold used to differentiate and classify low‐mobility (LM) and high‐mobility (HM) polygynous queens. Colony identity is provided on the x‐axis while queen mobility (estimated marginal mean for polygynous colonies, mean for monogynous queens) is provided on the y‐axis.

To investigate whether queens in polygynous colonies of the ant *S. pallipes* differed in their mobility, we assessed the distance travelled between frames (one frame per minute) from 455 min of video recording per colony. In total, we analysed the mobility of 89 queens: 84 were from 25 polygynous colonies, and five queens were from five monogynous colonies (Table S3). One queen (C5_Green) died during the experiment and, thus, was excluded from the analysis. We found differences in mobility among queens in 88% (22/25) of the polygynous colonies (Figure 1 and Table S4).

The estimated marginal means for polygynous queen mobility ranged from 1.1 mm to 15.9 mm. In addition, the average mobility of monogynous queens ranged from 1.6 mm to 3.8 mm. We used this information to determine a threshold of 4 mm for the estimated marginal mean to classify polygynous queens as either low‐mobility queens (LM‐polygynous queens) or high‐mobility queens (HM‐polygynous queens). Among the 22 polygynous colonies with significant differences in queen mobility, 15 colonies had at least one LM‐polygynous queen and one HM‐polygynous queen (Figure 1).

Prior to the assessment of mating status and ovarian development, one queen of each mobility category was selected from each polygynous colony, resulting in 30 queens from 15 polygynous colonies (Figure S2). The selected queens were further analysed for mating status, ovarian development, behaviour, and gene expression in the brain and fat body. Mating status and ovarian development were assessed for the non‐selected queens of these colonies as well, resulting in a total of 21 LM‐polygynous queens, 29 HM‐polygynous queens, and five monogynous queens examined.

### 3.2 Low‐Mobility Queens Were More Likely to be Mated Than High‐Mobility Queens

**FIGURE 2:**
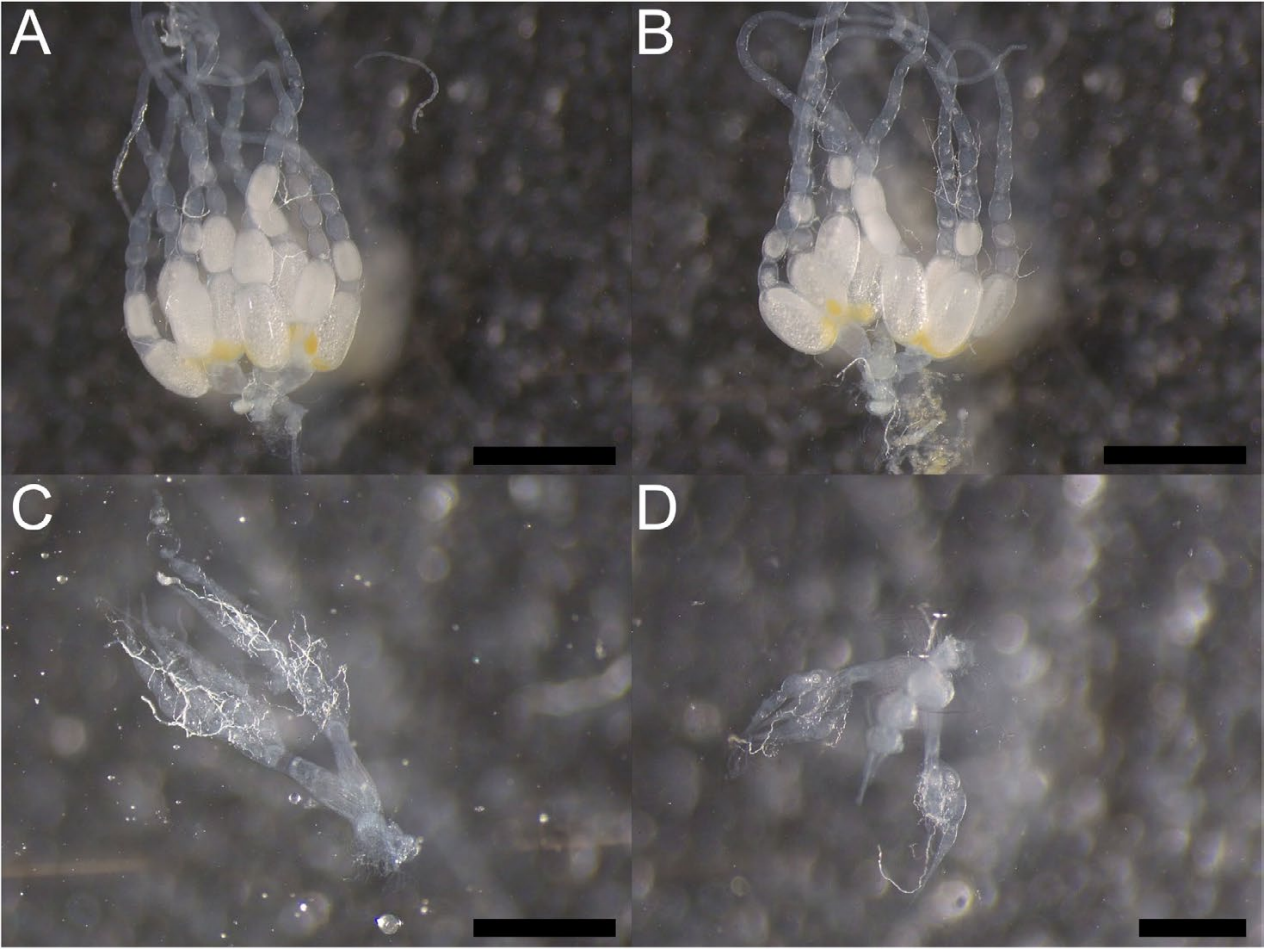
Ovarian development differed between queen types as well as workers in *Stigmatomma pallipes*. Representative images of the dissected ovaries of: (A) monogynous queens; (B) low‐mobility (LM) polygynous queens; (C) high‐mobility (HM) polygynous queens; and (D) workers. Black bars represent 1 mm (A–C) or 500 μm (D).

To investigate whether mobility was associated with reproductive activity, we assessed the mating status and ovarian development of all analysed queens (Table S5). For comparison, we included a single worker from each colony as a non‐mated control. Out of the 15 polygynous colonies examined, which included 21 LM‐queens and 29 HM‐queens, all but two colonies (M1 and M10) contained one queen with a filled spermatheca, indicative of the queen being mated. All monogynous queens were mated. We also observed variation among queens in ovarian development: some presented well‐developed ovaries with yellow bodies, indicative of recent egg laying activity, while other queens had underdeveloped ovaries that resembled those of workers (Figure 2).

To elucidate whether mating status differed between LM‐ and HM‐queens in polygynous colonies, we tested the association between mobility category and mating status. We found very strong evidence for an association between mobility and mating status (Fisher's exact test, *p* < 0.0001). More specifically, 13 out of the 21 LM‐polygynous queens were mated (61%), whereas none of the 29 HM‐polygynous queens were. We also found that the level of mobility was associated with the degree of ovarian development (Fisher's exact test, *p* < 0.0001). Mated LM‐queens also had developed ovaries (69%), while only two (7%) of the HM‐queens had developed ovaries. LM‐queens were substantially more likely to be mated and have developed ovaries compared to HM‐queens in polygynous colonies. Of the 30 queens from polygynous colonies chosen for further behavioural and molecular investigations, all 15 HM‐polygynous queens were unmated, and all but two (13%) had undeveloped ovaries. Of the 15 LM‐polygynous queens chosen, 13 (87%) were mated and had developed ovaries.

### 3.3 Behavioural Clustering of Queens Revealed Two Clusters Associated With Mating and Mobility

**FIGURE 3:**
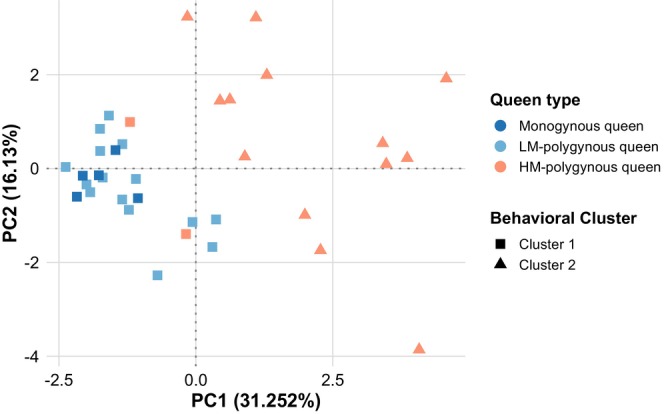
Principal Component Analysis revealed behavioural phenotypes of polygynous and monogynous queens associated with differences in mobility and mating status. A scatterplot displaying the first two principal components (PCs) from a principal component analysis (PCA) based on 13 scored behaviours for 35 queens. K‐means clustering based on all principal components revealed two clusters. The percentage of variance explained by each PC is provided in parentheses. Each point represents an individual queen with colours indicating individual queen types, while shapes denote behavioural cluster membership.

To investigate whether queen types differed in general terms of their overall behavioural phenotype, we scored 13 distinct behaviours (Table 1) every minute across five observation periods, for a total of 455 min. To examine general behavioural trends across queens, we applied a principal component analysis (PCA) using all observations and then performed *K*‐means clustering based on all principal components, identifying two clusters, which largely separated polygynous queens based on mobility (Fisher's exact test, *p* < 0.0001; Figure 3).

Cluster 1 consisted of all five monogynous queens (100%), all 15 LM‐polygynous queens (100%), and two out of the 15 HM‐polygynous queens (13%). These two queens had developed ovaries but were unmated (Table S5). Cluster 2 contained the remaining 13 out of 15 HM‐polygynous queens (87%).

### 3.4 Behavioural Differences Among Queens Were Driven by Resting, Self‐Grooming, and Walking

**FIGURE 4:**
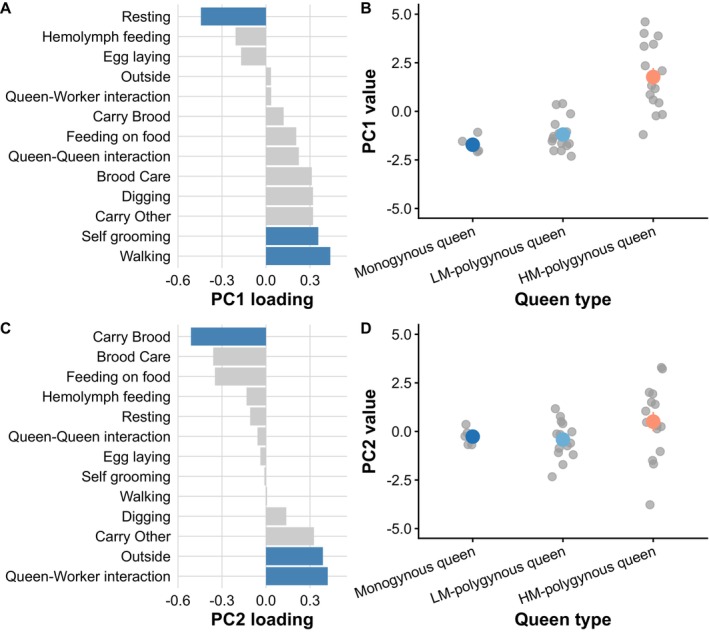
Loading of principal components revealed differences in behaviours associated with mobility in queens of *Stigmatomma pallipes*. (A) Principal component (PC) 1 captured variation in resting, self‐grooming, and walking (highlighted in blue). (B) There was strong evidence that PC1 values differed among queen types (*χ* ^2^ = 54.23, *p* < 0.0001). (C) PC2 captured variation in brood carrying, being outside, and interacting with workers (highlighted in blue). (D) There was weak evidence that PC2 values differed among queen types (*χ* ^2^ = 5.40, *p* = 0.067). Gray dots represent individual queens; coloured dots and error bars indicate mean ± SE for each queen type.

We next examined which behaviours differed the most between queen types by investigating the PCA loading of each behaviour. Collectively, eight PCs explained 90% of the variance. Based on loadings, PC1 was primarily associated with resting, self‐grooming, and walking behaviours (Figure 4A). PC1 values differed among queen types (LMM, *χ* ^2^ = 54.23, *p* < 0.0001; Figure 4B). Pairwise comparisons revealed that HM‐polygynous queens had higher PC1 values than monogynous (*t* = −5.16, *p* < 0.0001) and LM‐polygynous queens (*t* = 6.66, *p* < 0.0001), indicating they express more walking and self‐grooming behaviour and less resting. LM‐polygynous and monogynous queens did not differ based on PC1 (*t* = −0.78, *p* = 0.72). PC2 was positively associated with brood carrying behaviour and negatively correlated to being outside and expressing queen‐worker interactions (Figure 4C). We found weak evidence that queen types differed based on PC2 (LMM, *χ* ^2^ = 5.40, *p* = 0.067; Figure 4D). For PC3 to PC8, we found no evidence that queen types differed (all *p* > 0.1; Table S6).

### 3.5 Clustering of RNAseq Samples Revealed That Brain and Fat Body Transcriptomic Variation Was Associated With Mating and Mobility

**FIGURE 5:**
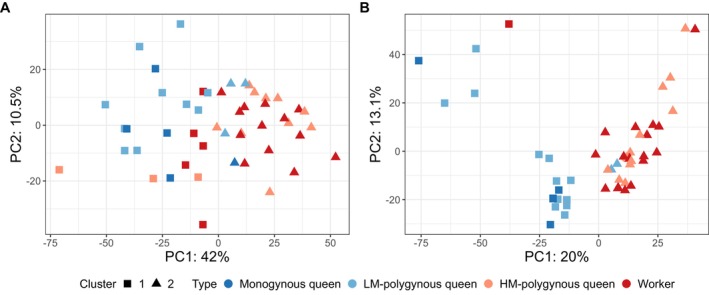
Brain and fat body transcriptomes showed variation associated with differences in mobility and mating status. Scatterplots displaying the first two principal components (PCs) from principal component analyses (PCA) based on: (A) brain; and (B) fat body transcriptomes for queens and workers. K‐means clustering analyses based on all PCs revealed two clusters for each tissue. Each point represents an individual with colours indicating individual types and shapes denoting cluster membership. The percentage of variance explained by each PC is provided.

We examined whether the brain and fat body transcriptomes were sufficient to group queens based on their mobility status. To do so, we conducted for each tissue a PCA on the full transcriptomes and performed K‐means clustering based on the principal components (Figure 5).

In the brain, the analysis identified two clusters (Figure 5A). Cluster 1 included four out of five (80%) monogynous queens and 10 out of 13 (77%) LM‐polygynous queens, as well as three out of 13 (23%) HM‐polygynous queens and five out of 18 (28%) workers. Cluster 2 contained one out of five monogynous queen (20%), three out of 13 LM‐polygynous queens (23%), 10 out of 13 HM‐polygynous queens (77%), and 13 out of 18 workers (72%). Cluster membership for polygynous queens was significantly associated with queen mobility (Fisher's exact test, *p* = 0.0169, Cramer's *V* = 0.538).

In the fat body, our analysis also revealed two clusters (Figure 5B). Cluster 1 contained all five monogynous queens, 12 out of 14 (86%) LM‐polygynous queens, one out of 15 (7%) HM‐polygynous queens, and one out of 19 (5%) workers. The one HM‐polygynous queen in Cluster 1 had developed ovaries. Cluster 2 comprised 13 out of 14 (93%) HM‐polygynous queens (93%) and 18 out of 19 workers (95%). In polygynous colonies, cluster membership was significantly associated with mobility (Fisher's exact test, *p* < 0.001, Cramer's *V* = 0.794).

### 3.6 Gene Expression Patterns of Monogynous and LM‐Polygynous Queens Differed From HM‐Polygynous Queens and Workers

To investigate whether the individual types differed in gene expression in brain and fat body, we quantified the number of differentially expressed genes (DEGs) between types. In the brain, we found 4456 DEGs among individual types (adjusted *p* < 0.05; 37% of the 11,871 tested genes). To identify general transcriptional patterns, we clustered DEGs based on expression profile, yielding six distinct expression groups (for cluster assignment of DEGs, see Table S7).

**FIGURE 6:**
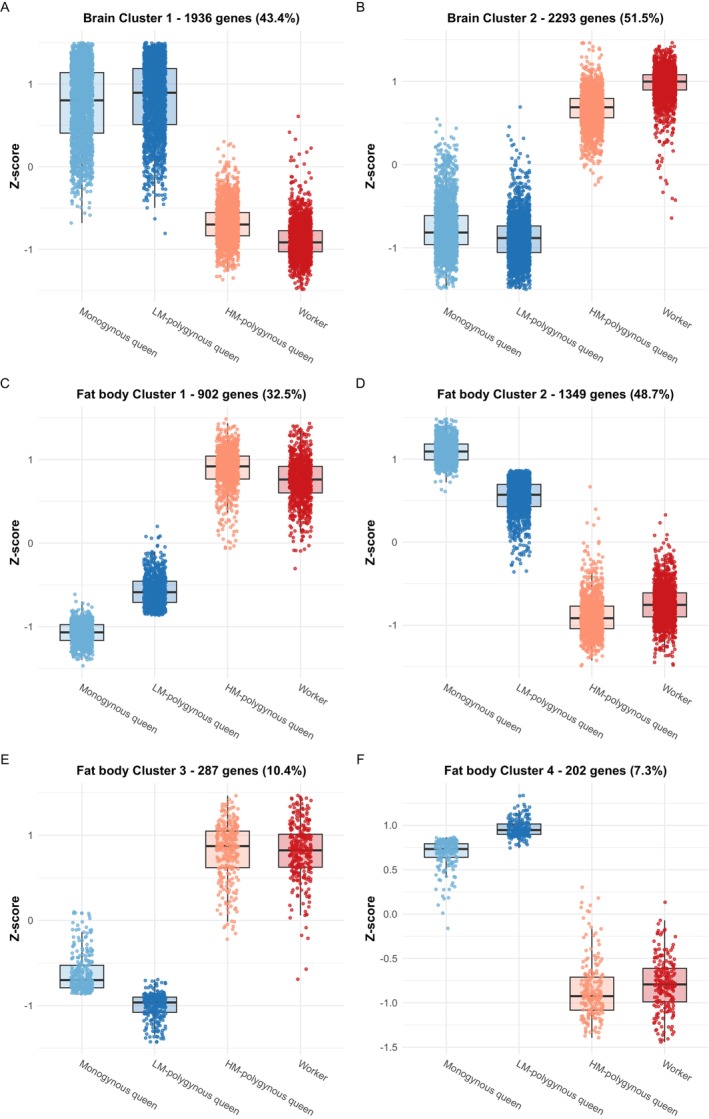
Clusters of differentially expressed genes (DEGs) separated mated from unmated individuals across (A, B) the brain and (C–F) the fat body. Each dot represents the *z*‐score‐transformed mean value of one gene, calculated from all individuals of that type. Coloured boxes and error bars indicate mean ± SE for each type.

The majority of DEGs (94%) were assigned to the first two clusters (Figure 6A,B): Cluster 1 (*n* = 1,936, 43% of DEGs) contained genes with higher relative expression in monogynous queens and LM‐polygynous queens compared to HM‐polygynous queens and workers; and cluster 2 (*n* = 2,293, 51% of DEGs) showed the opposite pattern. The remaining four clusters contained less than 5% of all DEGs (Figure S3A–D).

In the fat body, we found 2788 DEGs (adjusted *p* < 0.05, 23% of the 12,084 tested genes). Clustering of these DEGs based on expression produced five clusters, of which the first four accounted for 98% of all genes (Figure 6C–E). All clusters showed relative similarities between HM‐polygynous queens and workers, with both differing from monogynous and LM‐polygynous queens. Clusters differed in whether and how expression levels varied between monogynous and LM‐polygynous queens. Gene clusters 1 (*n* = 902, 32% of DEGs) and 4 (*n* = 202, 7% of DEGs) included genes with higher expression in LM‐polygynous queens relative to monogynous queens, whereas clusters 2 (*n* = 1,349, 48% of DEGs) and 3 (*n* = 287, 10% of DEGs) showed the opposite pattern. Cluster 5 only contained 1.2% of the DEGs (Figure S3E).

### 3.7 Genes That Differed in Expression Between LM‐ and HM‐Polygynous Queens Were Enriched for Translational and Neuronal Processes

To investigate the functional profiles of subsets of DEGs that shared similar expression differences among individual types, we performed Gene Ontology (GO) term enrichment analyses per cluster (Table 2).

**TABLE 2:** Functional enrichment based on gene ontology terms identified gene clusters in the brain and fat body to be enriched for translational and neurological terms. For each tissue, we provide the cluster number, the GO term ID, the GO term description, and the adjusted *p*‐value.

Tissue	Cluster	GO.ID	Description	Adjusted *p*
Brain	1	GO:0006418	tRNA aminoacylation for protein translation	0.046
		GO:0006364	rRNA processing	0.002
		GO:0032543	Mitochondrial translation	2.31e‐ 3
		GO:0002181	Cytoplasmic translation	1.08e‐15
	2	GO:0007268	Chemical synaptic transmission	0.039
		GO:0048010	Vascular endothelial growth factor receptor signalling pathway	0.037
		GO:0072659	Protein localization to plasma membrane	0.037
		GO:0008286	Insulin receptor signalling pathway	0.037
		GO:0045944	Positive regulation of transcription by RNA polymerase II	0.035
		GO:0007616	Long‐term memory	0.035
		GO:0008355	Olfactory learning	0.029
		GO:0050803	Regulation of synapse structure or activity	0.029
		GO:0045880	Positive regulation of smoothened signalling pathway	0.020
		GO:0032482	Rab protein signal transduction	0.014
		GO:0016319	Mushroom body development	0.013
		GO:0048190	Wing disc dorsal/ventral pattern formation	0.013
		GO:0007391	Dorsal closure	0.005
		GO:0006468	Protein phosphorylation	0.005
		GO:0090263	Positive regulation of canonical Wnt signalling pathway	0.005
		GO:0007173	Epidermal growth factor receptor signalling pathway	0.004
		GO:0008543	Fibroblast growth factor receptor signalling pathway	0.003
		GO:0007298	Border follicle cell migration	0.002
		GO:0007411	Axon guidance	0.002
		GO:0008587	Imaginal disc‐derived wing margin morphogenesis	0.002
Fat body	1	GO:0006120	Mitochondrial electron transport, NADH to ubiquinone	3.74e‐05
		GO:0032981	Mitochondrial respiratory chain complex I assembly	6.61e‐03
	2	GO:0006357	Regulation of transcription by RNA polymerase II	1.77e‐03

GO term enrichment analysis of cluster‐associated DEGs identified in the brain revealed significant enrichment for DEGs in clusters 1 and 2 (Table 2). DEGs in cluster 1, which had relatively higher expression in monogynous queens and LM‐polygynous queens, were enriched for four terms, including cytoplasmic translation (GO:0002181), mitochondrial translation (GO:0032543), rRNA processing (GO:0006364), and RNA aminoacylation for protein translation (GO:0006418). For DEGs within cluster 2, which were elevated in workers and HM‐polygynous queens (Figure 6), 20 enriched terms were identified, including axon guidance (GO:0007411), epidermal growth factor receptor signalling pathway (GO:0007173), mushroom body development (GO:0016319), and insulin receptor signalling pathway (GO:0008286).

In the fat body, similar to the brain, we detected enriched GO terms for DEGs in clusters 1 and 2 (Table 2). DEGs in cluster 1, which encompassed genes with higher expression in HM‐polygynous queens and workers, were enriched for two terms: mitochondrial electron transport, NADH to ubiquinone (GO:0006120) and mitochondrial respiratory chain complex I assembly (GO:0032981). DEGs in cluster 2, characterized by genes with higher expression in monogynous queens and LM‐polygynous queens, showed enrichment for a single term: Regulation of transcription by RNA polymerase II (GO:0006357).

## 4 Discussion

**FIGURE 7:**
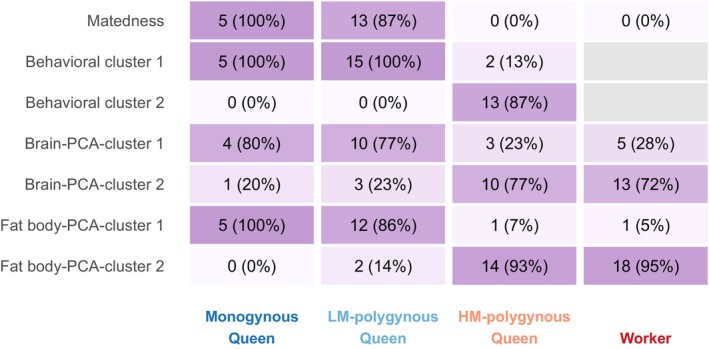
The relative distributions of mating status, behaviour‐based clusters, and transcriptome‐based clusters illustrate that low‐mobility polygynous queens resembled monogynous queens, while high‐mobility polygynous queens (actually dealated gynes) were similar to workers. A heatmap illustrating the proportion of individuals assigned to each cluster category for monogynous queens, low‐mobility (LM) polygynous queens, high‐mobility (HM) polygynous queens, and workers. Numbers denote the number of individuals in the respective category with the percentage compared to the overall number of individuals in that group provided in parenthesis. The colour gradient indicates the percentage of individuals within each group assigned to the respective category.

To better understand behavioural and physiological variation within the queen caste in polygynous colonies, we investigated the mobility, mating status, behaviour, and gene expression of queens in polygynous colonies of the ant *S. pallipes* and compared them to monogynous queens and workers. Across our analyses, two distinct polygynous queen types emerged: low‐mobility (LM) mated queens that shared characteristics with monogynous mated queens and high‐mobility (HM) unmated queens that grouped with workers on different biological scales ranging from the molecular to the organismal (Figure 7). These findings reveal that some queens in polygynous *S. pallipes* colonies are actually dealated gynes (unmated queens) that resemble workers in both their behavioural phenotype as well as the underlying molecular mechanisms.

The behavioural differences between the mated LM‐ and unmated HM‐queens were primarily driven by mobility‐related behaviours, such as resting, walking, and foraging outside the nest. LM‐polygynous and monogynous queens, which were mated and reproductively active, showed behavioural similarities to established queens in other social insect species, as the focus on reproduction is usually associated with reduced mobility (Free et al. 1992; Nagel et al. 2020). For example, *Harpegnathos saltator* mated workers move less and are more likely to stay inside the nest compared to unmated workers (Penick et al. 2021; Haight and Liebig 2025). Interestingly, within our analysis, LM‐polygynous and monogynous queens also engaged in interactions with the brood, which stands in contrast to the usual specialization of ant queens in egg laying when in the presence of workers (Majidifar et al. 2024). Previous behavioural observations of two *S. pallipes* queens from a single colony revealed a comparable behavioural repertoire (Traniello 1982) and queens have been reported to partake in upkeep of the colonies (Haskins 1928). The interactions with the brood may be explained by *S. pallipes* queens feeding on the regurgitated liquids of larvae (Haskins 1928; Traniello 1982).

The transcriptomic analyses were consistent with the behavioural analyses in that they revealed that LM‐polygynous queens and monogynous queens had similar brain and fat body gene expression patterns, which differed from HM‐polygynous queens and workers. This finding was supported by both the clustering of samples based on overall gene expression and the targeted analysis of the genes that showed expression differences among individual types. In both tissues, we found a large number of differentially expressed genes (DEGs) between LM‐ and HM‐polygynous queens. Previous transcriptomic studies that compared queens in different conditions, such as age and mating status, reported fewer differentially expressed genes (Lucas and Keller 2018; Nagel et al. 2020; Ferreira et al. 2025). Finding thousands of differentially expressed genes is usually restricted to comparisons between anatomically and morphologically differentiated castes (Grozinger et al. 2007; Harrison et al. 2015; Morandin et al. 2016).

The transcriptomic variation between LM‐ and HM‐polygynous queens may be partly explained by differences in mating status. Although this was not conducted in the context of polygyny, previous studies that compared unmated queens before colony foundation and established mated queens also reported large transcriptomic variation associated with the mating status (von Wyschetzki et al. 2015; Nagel et al. 2020; Liu et al. 2023). The age of the queens was also found to be associated with variation in gene expression in multiple ant species (von Wyschetzki et al. 2015; Lucas et al. 2017; Lucas and Keller 2018; Negroni et al. 2019; Nagel et al. 2020; Harrison et al. 2021). Therefore, the age of the queens used in our study may explain some of the transcriptomic variation. While their exact age remains unknown, the unmated queens may have been younger because they were observed to be adopted by established colonies with older, mated queens (Traniello 1982). In addition, other factors such as variation in nutrition and/or care received by the workers may have driven the transcriptomic differences between LM‐ and HM‐polygynous queens.

Differentially expressed genes between HM‐polygynous queens and workers compared to LM‐polygynous queens and monogynous queens showed tissue‐specific functional enrichment. In the brain, genes that were more highly expressed in HM‐polygynous queens and workers relative to LM‐polygynous queens and monogynous queens were enriched for axon guidance, insulin signaling, mushroom‐body development and synaptic signaling, which are expected to support navigation, sensory processing, and task flexibility (Ament et al. 2008; Alleman et al. 2019; Nagel et al. 2020). This is consistent with reports in other ant species, where gynes upregulated genes involved in mushroom body functioning and axon guidance (Nagel et al. 2020), and reproductive individuals showed reduced expression of genes involved in neural investment (Julian and Gronenberg 2002; Groh et al. 2006; Penick et al. 2021). Meanwhile, genes overexpressed in the brain of LM‐polygynous queens and monogynous queens showed the highest confidence for enrichment for processes related to cytoplasmic translation, which was also enriched in genes upregulated in the brain of mated queens compared to unmated queens in another ant species (Nagel et al. 2020). In the fat body, genes with higher expression in HM‐polygynous queens and workers were enriched for mitochondrial processes, which aligns with previous reports that insect fat bodies adjust mitochondrial content and activity in response to physiological demands (Arrese and Soulages 2010; Treidel et al. 2023). Finally, our finding of enhanced protein synthesis in the fat body of LM‐polygynous and monogynous queens may reflect physiological processes associated with egg production (Price 1973; Jensen and Børgesen 2000; Arrese and Soulages 2010) as both sets of queens were mostly reproductively active.

Our results provide strong evidence that most *S. pallipes* polygynous colonies from the population used in this study were actually monogynous, as they contained only one inseminated and reproductively active queen with the other queens being dealated gynes (unmated queens). This stands in contrast with a previous report of multiple reproductively active queens within *S. pallipes* colonies (Traniello 1982). However, this earlier finding was merely supported by behavioural observations and only concerned a subset of the observed colonies (Traniello 1982). As the colonies from that study were collected in Westford, Massachusetts in spring and summer 1977–1978 (Traniello 1982), and the colonies in our study were collected in the Huyck Preserve, New York in May 2024, it may also be that the social structure of *S. pallipes* differs among populations, as found in other ants (Gill et al. 2009), or that colonies with multiple reproductively active queens are rare. By focusing on colonies that showed mobility variation among queens, our analyses did not include four colonies that contained several queens with low mobility values (Figure S2). We cannot rule out that these four polygynous colonies may have contained multiple mated queens.

Several lines of evidence show that the unmated HM‐queens in polygynous *S. pallipes* colonies were analogous to workers at behavioural and molecular levels. First, they exhibited high mobility and more routinely expressed behaviours, such as foraging outside, digging, and transporting nest material, which represent typical worker behaviours. Such expression of non‐reproductive behaviours by queens was also reported in several species of ants, including *S. pallipes* , where gynes do not mate or disperse, and instead shed their wings and remain in their natal colony to perform tasks usually carried out by workers (Haskins 1928; Johnson et al. 2007; Nehring et al. 2012). Our finding also echoes the description of a worker‐like reproductive caste expressing worker‐like behaviours in the closely related polygynous species *Mystrium oberthueri* (Molet et al. 2007).

While previous reports of queens resembling workers were generally limited to behavioural description and the analysis of ovarian development (Buschinger 1968; Ito et al. 1996; Hora et al. 2005; Holbrook et al. 2007; Vieira et al. 2012; Nehring et al. 2012; Araújo et al. 2016; Murakami 2020), we demonstrate that the unmated HM‐queens in polygynous *S. pallipes* colonies had very similar transcriptomic profiles to workers, both in the brain and in the fat body. In contrast, they showed large differences in gene expression compared to mated queens from either monogynous or polygynous colonies. Interestingly, while queens and workers have very distinct transcriptomic profiles (Grozinger et al. 2007; Ferreira et al. 2013; Feldmeyer et al. 2014; Morandin et al. 2017; Das and de Bekker 2022), our analyses did not detect any brain nor fat body gene expression differences when collectively comparing the queen and worker castes, presumably because of the worker‐like gene expression patterns of HM‐polygynous queens.

Our study establishes measurements of mobility as a non‐invasive method to predict reproductive variation between queens in polygynous *S. pallipes* colonies, although future work will be needed to validate it in other ant species. This method opens the possibility for experimental manipulations of colony composition through the targeted removal of specific individuals to assess their influence on colony organization and reproductive dynamics. Such studies will contribute to a better understanding of how reproductive roles are regulated across polygynous systems.

The behaviour of social insect queens is traditionally viewed as more homogeneous than that of the workers because the role of queens in mature colonies is restricted to producing eggs while workers fulfil all non‐reproductive tasks. The behavioural heterogeneity of the worker caste is best illustrated by age polyethism, whereby younger workers tend to perform intranidal tasks, such as brood care, while older workers tend to leave the nest and forage for food (Tripet and Nonacs 2004; Winston 1991; Camargo et al. 2007; Wilson 1976; Seeley 1982; Beshers and Traniello 1996). This behavioural transition is associated with transcriptomic and physiological changes (Kohlmeier et al. 2019; Dolezal et al. 2013; Kühbandner et al. 2014). Here, we report large behavioural and transcriptomic variation within the queen caste, with the coexistence in polygynous *S. pallipes* colonies of mated queens that monopolize egg production and dealated gynes that are characterized by worker‐like behavioural and gene expression. Our results, together with recent reports of context‐dependent queen specialization (Majidifar et al. 2024; Woodard et al. 2013), raise questions on the constitutive specialization in egg production of social insect queens and call for further studies of variation within the queen caste at multiple phenotypic levels to better understand division of labor in insect societies.

## Author Contributions

M.F.B. conceived the idea and designed the methodology together with R.L. and T.J.C. M.F.B. collected the ants and conducted the experiments. M.F.B. and R.L. analysed the data. J.S.M. and T.J.C. assembled and annotated the genome. M.F.B., R.L., and T.J.C. led the writing of the manuscript. All authors contributed critically to the drafts and gave final approval for publication.

## Funding

This project was funded by the Deutsche Forschungsgemeinschaft (DFG, German Research Foundation) GRK2526/1 Project number: 407023052 (TJC and RL), Agence Nationale de la Recherche (ANR, French Research Agency) ANR‐23‐CE02‐0004 (project ANTOGENY) (RL), Deutsche Bundesstiftung Umwelt (DBU, German Federal Environmental Foundation) 20021/743 (JSM), and with support from a Huyck Research Grant 2024 (MFB).

## Disclosure

Raw sequence data have been uploaded to the NCBI Sequence Read Archive (BioProject: PRJNA1371108). The assembled genome and annotations are hosted on a publicly accessible GitHub repository: https://github.com/Joscolgan/stigmatomma_pallipes_genome.

## Conflicts of Interest

The authors declare no conflicts of interest.

## Supporting information


**Figure S1:** Limited variation across observations in queen behaviour. The scatterplot displays the first two principal components from a principal component analysis (PCA) based on 13 scored behaviours for 35 queens across the five observations. The percentage of variance explained by each PC is provided in parentheses. Ellipses represent 95% confidence intervals around group centroids, colours indicate different observations, small dots correspond to individual queens, large dots to the group centroid.
**Figure S2:** Mobility variation among *Stigmatomma pallipes* queens from monogynous and polygynous colonies allowed the selection of samples for further analyses. The dashed blue line at 4 mm indicates the threshold used to differentiate low mobility (LM) and high mobility (HM) polygynous queens. Indicated as green triangles are queens that were selected for mating status, ovarian development, behaviour, and gene expression in the brain and fat body. Colony identity is provided on the x‐axis, while queen mobility (estimated marginal mean for polygynous, mean for monogynous queens) is provided on the y‐axis.
**Figure S3:** Clusters of differentially expressed genes (DEGs) that show similar expression differences across individual types for (A–D) the brain and (E) the fat body. Each cluster represented less than 5% of the number of DEGs. Each dot represents the *z*‐score‐transformed mean value of one gene, calculated from all individuals of that category.

**Table S1:** Summary table of the colonies used in the study, providing the number of queens and workers per colony.
**Table S2:** RNA sequencing depth of brain and fat body samples.
**Table S3:** Mobility values of *Stigmatomma pallipes* queens from 30 different colonies.
**Table S4:** Summary outputs of ANOVAs to test mobility differences among queens within each polygynous colony. *p* values below the 5% threshold are indicated in bold.
**Table S5:** Output of the examination of reproductive activity in *S.pallipes* queens and workers.
**Table S6:** Outputs of ANOVAs to test the effect of queen type on the principal components extracted from the PCA based on the full behavioural dataset. *p* values below the 5% threshold are indicated in bold.
**Table S7:** Cluster assignment of differentially expressed genes in brain and fat body.

**Data S1:** mec70360‐sup‐0003‐Supinfo.zip.

## Data Availability

All raw data used in this study are available as part of the Supporting Information—S1 or made publicly available from the NCBI Sequence Read Archive (BioProject: PRJNA1371108). The code and input data for all analyses are provided as Supporting Information—S1.

## References

[mec70360-bib-0118] Adrian Alexa, J. R. 2017. “topGO. Bioconductor.” 10.18129/B9.BIOC.TOPGO.

[mec70360-bib-0002] Alleman, A. , M. Stoldt , B. Feldmeyer , and S. Foitzik . 2019. “Tandem‐Running and Scouting Behaviour Are Characterized by Up‐Regulation of Learning and Memory Formation Genes Within the Ant Brain.” Molecular Ecology 28, no. 9: 2342–2359. 10.1111/mec.15079. 30903719

[mec70360-bib-0003] Ament, S. A. , M. Corona , H. S. Pollock , and G. E. Robinson . 2008. “Insulin Signaling Is Involved in the Regulation of Worker Division of Labor in Honey Bee Colonies.” Proceedings of the National Academy of Sciences 105, no. 11: 4226–4231. 10.1073/pnas.0800630105. PMC2393790 18337502

[mec70360-bib-0004] Andrews, S. 2010. A Quality Control Tool for High Throughput Sequence Data. *Fastqc* . http://www.bioinformatics.babraham.ac.uk/projects/fastqc/.

[mec70360-bib-0005] Araújo, M. d. S. , J. P. R. Oliveira , D. J. de Souza , M. A. Oliveira , and F. G. de Jesus . 2016. “Castas Sexuadas de Trachymyrmex Fuscus (Formicidae: Attini) Executando Tarefas de Operárias.” Ciência Rural 46: 199–202. 10.1590/0103-8478cr20150189.

[mec70360-bib-0006] Arrese, E. L. , and J. L. Soulages . 2010. “Insect Fat Body: Energy, Metabolism, and Regulation.” Annual Review of Entomology 55: 207–225. 10.1146/annurev-ento-112408-085356. PMC3075550 19725772

[mec70360-bib-0007] Bates, D. , M. Maechler , B. Bolker , and S. Walker . 2003. “Lme4: Linear Mixed‐Effects Models Using “Eigen” and S4.” 10.32614/CRAN.package.lme4.

[mec70360-bib-0008] Berens, A. J. , J. H. Hunt , and A. L. Toth . 2015. “Comparative Transcriptomics of Convergent Evolution: Different Genes but Conserved Pathways Underlie Caste Phenotypes Across Lineages of Eusocial Insects.” Molecular Biology and Evolution 32, no. 3: 690–703. 10.1093/molbev/msu330. 25492498

[mec70360-bib-0009] Beshers, S. N. , and J. F. A. Traniello . 1996. “Polyethism and the Adaptiveness of Worker Size Variation in the Attine Septentrionalis.” Journal of Insect Behavior 9, no. 1: 61–83. 10.1007/BF02213724.

[mec70360-bib-0010] Bonasio, R. , G. Zhang , C. Ye , et al. 2010. “Genomic Comparison of the Ants Camponotus Floridanus and *Harpegnathos saltator* .” Science 329, no. 5995: 1068–1071. 10.1126/science.1192428. 20798317 PMC3772619

[mec70360-bib-0011] Boomsma, J. J. , and R. Gawne . 2018. “Superorganismality and Caste Differentiation as Points of no Return: How the Major Evolutionary Transitions Were Lost in Translation.” Biological Reviews 93, no. 1: 1–54. 10.1111/brv.12330. 28508537

[mec70360-bib-0012] Boomsma, J. J. , D. B. Huszár , and J. S. Pedersen . 2014. “The Evolution of Multiqueen Breeding in Eusocial Lineages With Permanent Physically Differentiated Castes.” Animal Behaviour 92: 241–252. 10.1016/j.anbehav.2014.03.005.

[mec70360-bib-0013] Bray, N. L. , H. Pimentel , P. Melsted , and L. Pachter . 2016. “Near‐Optimal Probabilistic RNA‐Seq Quantification.” Nature Biotechnology 34, no. 5: 525–527. 10.1038/nbt.3519. 27043002

[mec70360-bib-0014] Brown, M. J. F. , and S. Bonhoeffer . 2003. “On the Evolution of Claustral Colony Founding in Ants.” Evolutionary Ecology Research 5, no. 2: 305–313.

[mec70360-bib-0015] Buschinger, A. 1968. “Mono‐ Und Polygynie Bei Arten der gattungLeptothorax Mayr (Hymenoptera Formicidæ).” Insectes Sociaux 15, no. 3: 217–225. 10.1007/BF02225844.

[mec70360-bib-0016] Camargo, R. S. , L. C. Forti , J. F. S. Lopes , A. P. P. Andrade , and A. L. T. Ottati . 2007. “Age Polyethism in the Leaf‐Cutting Ant *Acromyrmex Subterraneus Brunneus* Forel, 1911 (Hym., Formicidae).” Journal of Applied Entomology 131, no. 2: 139–145. 10.1111/j.1439-0418.2006.01129.x.

[mec70360-bib-0017] Caminer, M. A. , R. Libbrecht , M. Majoe , D. V. Ho , P. Baumann , and S. Foitzik . 2023. “Task‐Specific Odorant Receptor Expression in Worker Antennae Indicates That Sensory Filters Regulate Division of Labor in Ants.” Communications Biology 6, no. 1: 1004. 10.1038/s42003-023-05273-4. 37783732 PMC10545721

[mec70360-bib-0117] Carlson, M. 2017. “GO.db. Bioconductor.” 10.18129/B9.BIOC.GO.DB.

[mec70360-bib-0018] Chen, S. , Y. Zhou , Y. Chen , and J. Gu . 2018. “Fastp: An Ultra‐Fast All‐In‐One FASTQ Preprocessor.” Bioinformatics 34, no. 17: i884–90. 10.1093/bioinformatics/bty560. 30423086 PMC6129281

[mec70360-bib-0019] Corona, M. , R. Libbrecht , and D. E. Wheeler . 2016. “Molecular Mechanisms of Phenotypic Plasticity in Social Insects.” In Current Opinion in Insect Science, vol. 13, 55–60. Insect genomics * Development and regulation. 10.1016/j.cois.2015.12.003. 27436553

[mec70360-bib-0020] Corona, M. , R. Libbrecht , Y. Wurm , O. Riba‐Grognuz , R. A. Studer , and L. Keller . 2013. “Vitellogenin Underwent Subfunctionalization to Acquire Caste and Behavioral Specific Expression in the Harvester Ant *Pogonomyrmex barbatus* .” PLoS Genetics 9, no. 8: e1003730. 10.1371/journal.pgen.1003730. 23966882 PMC3744404

[mec70360-bib-0021] Das, B. , and C. de Bekker . 2022. “Time‐Course RNASeq of *Camponotus floridanus* Forager and Nurse Ant Brains Indicate Links Between Plasticity in the Biological Clock and Behavioral Division of Labor.” BMC Genomics 23, no. 1: 57. 10.1186/s12864-021-08282-x. 35033027 PMC8760764

[mec70360-bib-0022] de Coster, W. , and R. Rademakers . 2023. “NanoPack2: Population‐Scale Evaluation of Long‐Read Sequencing Data.” Bioinformatics 39, no. 5: btad311. 10.1093/bioinformatics/btad311. 37171891 PMC10196664

[mec70360-bib-0023] Dolezal, A. G. , J. Johnson , B. Hölldobler , and G. V. Amdam . 2013. “Division of Labor Is Associated With Age‐Independent Changes in Ovarian Activity in *Pogonomyrmex californicus* Harvester Ants.” Journal of Insect Physiology 59, no. 4: 519–524. 10.1016/j.jinsphys.2013.02.008. 23473699

[mec70360-bib-0024] Emms, D. M. , and S. Kelly . 2015. “OrthoFinder: Solving Fundamental Biases in Whole Genome Comparisons Dramatically Improves Orthogroup Inference Accuracy.” Genome Biology 16, no. 1: 157. 10.1186/s13059-015-0721-2. 26243257 PMC4531804

[mec70360-bib-0025] Evans, L. J. , and N. E. Raine . 2014. “Changes in Learning and Foraging Behaviour Within Developing Bumble Bee ( *Bombus terrestris* ) Colonies.” PLoS One 9, no. 3: e90556. 10.1371/journal.pone.0090556. 24599144 PMC3943973

[mec70360-bib-0026] Ewels, P. , M. Magnusson , S. Lundin , and M. Käller . 2016. “MultiQC: Summarize Analysis Results for Multiple Tools and Samples in a Single Report.” Bioinformatics 32, no. 19: 3047–3048. 10.1093/bioinformatics/btw354. 27312411 PMC5039924

[mec70360-bib-0027] Feldmeyer, B. , D. Elsner , and S. Foitzik . 2014. “Gene Expression Patterns Associated With Caste and Reproductive Status in Ants: Worker‐Specific Genes Are More Derived Than Queen‐Specific Ones.” Molecular Ecology 23, no. 1: 151–161. 10.1111/mec.12490. 24118315

[mec70360-bib-0028] Ferreira, P. G. , S. Patalano , R. Chauhan , et al. 2013. “Transcriptome Analyses of Primitively Eusocial Wasps Reveal Novel Insights Into the Evolution of Sociality and the Origin of Alternative Phenotypes.” Genome Biology 14, no. 2: R20. 10.1186/gb-2013-14-2-r20. 23442883 PMC4053794

[mec70360-bib-0029] Ferreira, T. N. , M.‐E. Chen , P. Saelao , C. Tamborindeguy , and P. V. Pietrantonio . 2025. “Fire Ant Ovary Gene Expression Analyses Revealed Immune and Insulin Pathways Underlie the Reproductive Transition From Virgin to Mated Queen.” BMC Genomics 26, no. 1: 735. 10.1186/s12864-025-11900-7. 40781584 PMC12333097

[mec70360-bib-0030] FFmpeg developers . 2024. FFMPEG. https://ffmpeg.org/.

[mec70360-bib-0031] Fox, J. , S. Weisberg , and B. Price . 2001. “Car: Companion to Applied Regression.” 10.32614/CRAN.package.car.

[mec70360-bib-0032] Free, J. B. , A. W. Ferguson , and J. R. Simpkins . 1992. “The Behaviour of Queen Honeybees and Their Attendants.” Physiological Entomology 17, no. 1: 43–55. 10.1111/j.1365-3032.1992.tb00988.x.

[mec70360-bib-0116] Friard, O. , and M. Gamba . 2016. “BORIS: A Free, Versatile Open‐Source Event‐Logging Software for Video/Audio Coding and Live Observations.” Methods in Ecology and Evolution 7, no. 11: 1325–1330. 10.1111/2041-210x.12584.

[mec70360-bib-0119] Gabriel, L. , T. Brůna , K. J. Hoff , et al. 2024. “BRAKER3: Fully Automated Genome Annotation Using RNA‐Seq and Protein Evidence With GeneMark‐ETP, AUGUSTUS, and TSEBRA.” Genome Research 34, no. 5: 769–777. 10.1101/gr.278090.123. 38866550 PMC11216308

[mec70360-bib-0034] Gadagkar, R. 1997. “The Evolution of Caste Polymorphism in Social Insects:0 Genetic Release Followed by Diversifying Evolution.” Journal of Genetics 76, no. 3: 167–179. 10.1007/BF02932215.

[mec70360-bib-0115] Gadau, J. , and J. Fewell , eds. 2009. Organization of Insect Societies. Harvard University Press. 10.2307/j.ctv228vr0t.

[mec70360-bib-0035] Gill, R. J. , A. Arce , L. Keller , and R. L. Hammond . 2009. “Polymorphic Social Organization in an Ant.” Proceedings of the Royal Society B: Biological Sciences 276, no. 1677: 4423–4431. 10.1098/rspb.2009.1408. PMC2817112 19793758

[mec70360-bib-0036] Groh, C. , D. Ahrens , and W. Rössler . 2006. “Environment‐ and Age‐Dependent Plasticity of Synaptic Complexes in the Mushroom Bodies of Honeybee Queens.” Brain, Behavior and Evolution 68, no. 1: 1–14. 10.1159/000092309. 16557021

[mec70360-bib-0037] Grozinger, C. M. , Y. Fan , S. E. R. Hoover , and M. L. Winston . 2007. “Genome‐Wide Analysis Reveals Differences in Brain Gene Expression Patterns Associated With Caste and Reproductive Status in Honey Bees ( *Apis mellifera* ).” Molecular Ecology 16, no. 22: 4837–4848. 10.1111/j.1365-294X.2007.03545.x. 17927707

[mec70360-bib-0038] Gurevich, A. , V. Saveliev , N. Vyahhi , and G. Tesler . 2013. “QUAST: Quality Assessment Tool for Genome Assemblies.” Bioinformatics 29, no. 8: 1072–1075. 10.1093/bioinformatics/btt086. 23422339 PMC3624806

[mec70360-bib-0120] Haight, K. L. , and J. Liebig . 2025. “Reduced Movement Activity in Reproductive Workers of the ant *Harpegnathos saltator* as Part of a Social Insect Reproductive Syndrome.” Insectes Sociaux 73, no. 1: 129–136. 10.1007/s00040-025-01052-y.

[mec70360-bib-0040] Harrison, M. C. , R. L. Hammond , and E. B. Mallon . 2015. “Reproductive Workers Show Queenlike Gene Expression in an Intermediately Eusocial Insect, the Buff‐Tailed Bumble Bee *Bombus terrestris* .” Molecular Ecology 24, no. 12: 3043–3063. 10.1111/mec.13215. 25913260

[mec70360-bib-0041] Harrison, M. C. , L. M. Jaimes Niño , M. Almeida Rodrigues , et al. 2021. “Gene Coexpression Network Reveals Highly Conserved, Well‐Regulated Anti‐Ageing Mechanisms in Old Ant Queens.” Genome Biology and Evolution 13, no. 6: evab093. 10.1093/gbe/evab093. 33944936 PMC8214412

[mec70360-bib-0042] Haskins, C. P. 1928. “Notes on the Behavior and Habits of Stigmatomma Pallipes Haldeman.” Journal of the New York Entomological Society 36, no. 2: 179–184.

[mec70360-bib-0043] Haskins, C. P. , and E. V. Enzmann . 1937. “Studies of Certain Sociological and Physiological Features in the Formicidae.” Annals of the New York Academy of Sciences 37, no. 1: 97–162. 10.1111/j.1749-6632.1937.tb55482.x.

[mec70360-bib-0044] Hellemans, S. , and R. Hanus . 2024. “Termite Primary Queen—Ancestral, but Highly Specialized Eusocial Phenotype.” Current Opinion in Insect Science 61: 101157. 10.1016/j.cois.2023.101157. 38142979

[mec70360-bib-0045] Holbrook, C. T. , C.‐P. Strehl , R. A. Johnson , and J. Gadau . 2007. “Low Queen Mating Frequency in the Seed‐Harvester Ant Pogonomyrmex (Ephebomyrmex) Pima: Implications for the Evolution of Polyandry.” Behavioral Ecology and Sociobiology 62, no. 2: 229–236.

[mec70360-bib-0113] Hölldobler, B. , and E. O. Wilson . 1977. “The Number of Queens: An Important Trait in Ant Evolution.” Naturwissenschaften 64, no. 1: 8–15.

[mec70360-bib-0046] Hora, R. R. , E. Vilela , R. Fénéron , A. Pezon , D. Fresneau , and J. Delabie . 2005. “Facultative Polygyny in *Ectatomma tuberculatum* (Formicidae, Ectatomminae).” Insectes Sociaux 52, no. 2: 194–200. 10.1007/s00040-004-0794-5.

[mec70360-bib-0047] Huang, N. , and H. Li . 2023. “Compleasm: A Faster and More Accurate Reimplementation of BUSCO.” Bioinformatics 39, no. 10: btad595. 10.1093/bioinformatics/btad595. 37758247 PMC10558035

[mec70360-bib-0048] Ito, F. , N. R. Yusoff , and A. H. Idris . 1996. “Colony Composition and Queen Behavior in Polygynous Colonies of the Oriental Ponerine antOdontomachus Rixosus (Hymenoptera Formicidae).” Insectes Sociaux 43, no. 1: 77–86. 10.1007/BF01253958.

[mec70360-bib-0049] Jensen, P. V. , and L. W. Børgesen . 2000. “Regional and Functional Differentiation in the Fat Body of Pharaoh's Ant Queens, *Monomorium pharaonis* (L.).” Arthropod Structure & Development 29, no. 2: 171–184. 10.1016/S1467-8039(00)00021-9. 18088925

[mec70360-bib-0050] Johnson, R. A. , C. T. Holbrook , C. Strehl , and J. Gadau . 2007. “Population and Colony Structure and Morphometrics in the Queen Dimorphic Harvester Ant, *Pogonomyrmex pima* .” Insectes Sociaux 54, no. 1: 77–86. 10.1007/s00040-007-0916-y.

[mec70360-bib-0051] Julian, G. E. , and W. Gronenberg . 2002. “Reduction of Brain Volume Correlates With Behavioral Changes in Queen Ants.” Brain, Behavior and Evolution 60, no. 3: 152–164. 10.1159/000065936. 12417820

[mec70360-bib-0052] Kapheim, K. M. , H. Pan , C. Li , et al. 2015. “Genomic Signatures of Evolutionary Transitions From Solitary to Group Living.” Science 348, no. 6239: 1139–1143. 10.1126/science.aaa4788. 25977371 PMC5471836

[mec70360-bib-0114] Keller, L. 1991. “Queen Number, Mode of Colony Founding, and Queen Reproductive Success in Ants (Hymenoptera Formicidae).” Ethology Ecology & Evolution 3, no. 4: 307–316. 10.1080/08927014.1991.9525359.

[mec70360-bib-0053] Kim, D. , J. M. Paggi , C. Park , C. Bennett , and S. L. Salzberg . 2019. “Graph‐Based Genome Alignment and Genotyping With HISAT2 and HISAT‐Genotype.” Nature Biotechnology 37, no. 8: 907–915. 10.1038/s41587-019-0201-4. PMC7605509 31375807

[mec70360-bib-0054] Kohlmeier, P. , A. R. Alleman , R. Libbrecht , S. Foitzik , and B. Feldmeyer . 2019. “Gene Expression Is More Strongly Associated With Behavioural Specialization Than With Age or Fertility in Ant Workers.” Molecular Ecology 28, no. 3: 658–670. 10.1111/mec.14971. 30525254

[mec70360-bib-0055] Kolmogorov, M. , J. Yuan , Y. Lin , and P. A. Pevzner . 2019. “Assembly of Long, Error‐Prone Reads Using Repeat Graphs.” Nature Biotechnology 37, no. 5: 540–546. 10.1038/s41587-019-0072-8. 30936562

[mec70360-bib-0056] Korb, J. , K. Meusemann , D. Aumer , et al. 2021. “Comparative Transcriptomic Analysis of the Mechanisms Underpinning Ageing and Fecundity in Social Insects.” Philosophical Transactions of the Royal Society, B: Biological Sciences 376, no. 1823: 20190728. 10.1098/rstb.2019.0728. PMC7938167 33678016

[mec70360-bib-0057] Kühbandner, S. , A. P. Modlmeier , and S. Foitzik . 2014. “Age and Ovarian Development Are Related to Worker Personality and Task Allocation in the Ant *Leptothorax acervorum* .” Current Zoology 60, no. 3: 392–400. 10.1093/czoolo/60.3.392.

[mec70360-bib-0058] Kuznetsov, D. , F. Tegenfeldt , M. Manni , et al. 2023. “OrthoDB V11: Annotation of Orthologs in the Widest Sampling of Organismal Diversity.” Nucleic Acids Research 51, no. D1: D445–D451. 10.1093/nar/gkac998. 36350662 PMC9825584

[mec70360-bib-0059] Lenoir, A. , and A. Dejean . 1994. “Semi‐Claustral Colony Foundation in the Formicine Ants of the genusPolyrhachis (Hymenoptera: Formicidae).” Insectes Sociaux 41, no. 3: 225–234. 10.1007/BF01242293.

[mec70360-bib-0060] Lenth, R. V. 2025. Emmeans: Estimated Marginal Means, Aka Least‐Squares Means. https://CRAN.R‐project.org/package=emmeans.

[mec70360-bib-0061] Li, H. 2018. “Minimap2: Pairwise Alignment for Nucleotide Sequences.” Bioinformatics 34, no. 18: 3094–3100. 10.1093/bioinformatics/bty191. 29750242 PMC6137996

[mec70360-bib-0062] Libbrecht, R. , P. R. Oxley , and D. J. C. Kronauer . 2018. “Clonal Raider Ant Brain Transcriptomics Identifies Candidate Molecular Mechanisms for Reproductive Division of Labor.” BMC Biology 16, no. 1: 89. 10.1186/s12915-018-0558-8. 30103762 PMC6090591

[mec70360-bib-0063] Liu, F. , F. Xu , Y. Zhang , et al. 2023. “Comparative Analyses of Reproductive Caste Types Reveal Vitellogenin Genes Involved in Queen Fertility in *Solenopsis invicta* .” International Journal of Molecular Sciences 24, no. 24: 17130. 10.3390/ijms242417130. 38138959 PMC10743176

[mec70360-bib-0064] Love, M. I. , W. Huber , and S. Anders . 2014. “Moderated Estimation of Fold Change and Dispersion for RNA‐Seq Data With DESeq2.” Genome Biology 15, no. 12: 550. 10.1186/s13059-014-0550-8. 25516281 PMC4302049

[mec70360-bib-0065] Lucas, E. R. , and L. Keller . 2018. “Elevated Expression of Ageing and Immunity Genes in Queens of the Black Garden Ant.” Experimental Gerontology 108: 92–98. 10.1016/j.exger.2018.03.020. 29625209

[mec70360-bib-0066] Lucas, E. R. , J. Romiguier , and L. Keller . 2017. “Gene Expression Is More Strongly Influenced by Age Than Caste in the Ant *Lasius niger* .” Molecular Ecology 26, no. 19: 5058–5073. 10.1111/mec.14256. 28742933

[mec70360-bib-0067] Majidifar, V. , M. N. Psalti , M. Coulm , et al. 2024. “Ontogeny of Superorganisms: Social Control of Queen Specialization in Ants.” Functional Ecology 38, no. 5: 1044–1060. 10.1111/1365-2435.14536.

[mec70360-bib-0068] Manni, M. , M. R. Berkeley , M. Seppey , and E. M. Zdobnov . 2021. “BUSCO: Assessing Genomic Data Quality and Beyond.” Current Protocols 1, no. 12: e323. 10.1002/cpz1.323. 34936221

[mec70360-bib-0069] Mikheyev, A. S. , and T. A. Linksvayer . 2015. “Genes Associated With Ant Social Behavior Show Distinct Transcriptional and Evolutionary Patterns.” eLife 4: e04775. 10.7554/eLife.04775. 25621766 PMC4383337

[mec70360-bib-0070] Miura, T. , K. Oguchi , H. Yamaguchi , et al. 2022. “Understanding of Superorganisms: Collective Behavior, Differentiation and Social Organization.” Artificial Life and Robotics 27, no. 2: 204–212. 10.1007/s10015-022-00754-x.

[mec70360-bib-0071] Miyazaki, S. , H. Shimoji , R. Suzuki , et al. 2021. “Expressions of Conventional Vitellogenin and Vitellogenin‐Like A in Worker Brains Are Associated With a Nursing Task in a Ponerine Ant.” Insect Molecular Biology 30, no. 1: 113–121. 10.1111/imb.12682. 33150669

[mec70360-bib-0072] Mizumoto, N. , G. H. Gile , and S. C. Pratt . 2021. “Behavioral Rules for Soil Excavation by Colony Founders and Workers in Termites.” Annals of the Entomological Society of America 114, no. 5: 654–661. 10.1093/aesa/saaa017.

[mec70360-bib-0073] Molet, M. , C. Peeters , I. Follin , and B. L. Fisher . 2007. “Reproductive Caste Performs Intranidal Tasks Instead of Workers in the Ant *Mystrium oberthueri* .” Ethology 113, no. 7: 721–729. 10.1111/j.1439-0310.2007.01376.x.

[mec70360-bib-0074] Morandin, C. , A. S. Mikheyev , J. S. Pedersen , and H. Helanter . 2017. “Evolutionary Constraints Shape Caste‐Specific Gene Expression Across 15 Ant Species.” Evolution 71, no. 5: 1273–1284. 28262920 10.1111/evo.13220

[mec70360-bib-0075] Morandin, C. , M. M. Y. Tin , S. Abril , et al. 2016. “Comparative Transcriptomics Reveals the Conserved Building Blocks Involved in Parallel Evolution of Diverse Phenotypic Traits in Ants.” Genome Biology 17: 43. 10.1186/s13059-016-0902-7. 26951146 PMC4780134

[mec70360-bib-0076] Murakami, T. 2020. “Non‐Inseminated Queens Have Worker‐Like Behaviors in Colonies of Fungus‐Growing Ants, Mycetomoellerius Turrifex Wheeler (Attini, Hymenoptera).” Sociobiology 67, no. 3: 358–363. 10.13102/sociobiology.v67i3.5773.

[mec70360-bib-0077] Muratore, I. B. , S. P. Mullen , and J. F. A. Traniello . 2023. “Transcriptomic Analysis of Mosaic Brain Differentiation Underlying Complex Division of Labor in a Social Insect.” Journal of Comparative Neurology 531, no. 8: 853–865. 10.1002/cne.25469. 36895095

[mec70360-bib-0078] Nagel, M. , B. Qiu , L. E. Brandenborg , et al. 2020. “The Gene Expression Network Regulating Queen Brain Remodeling After Insemination and Its Parallel Use in Ants With Reproductive Workers.” Science Advances 6, no. 38: 1–17. 10.1126/sciadv.aaz5772. PMC7494347 32938672

[mec70360-bib-0079] Negroni, M. A. , S. Foitzik , and B. Feldmeyer . 2019. “Long‐Lived Temnothorax Ant Queens Switch From Investment in Immunity to Antioxidant Production With Age.” Scientific Reports 9, no. 1: 7270. 10.1038/s41598-019-43796-1. 31086243 PMC6514213

[mec70360-bib-0080] Nehring, V. , J. J. Boomsma , and P. d'Ettorre . 2012. “Wingless Virgin Queens Assume Helper Roles in Acromyrmex Leaf‐Cutting Ants.” Current Biology 22, no. 17: R671–R673. 10.1016/j.cub.2012.06.038. 22974989

[mec70360-bib-0081] Nie, H. , X. Shupeng , C. Xie , et al. 2018. “Comparative Transcriptome Analysis of *Apis mellifera* Antennae of Workers Performing Different Tasks.” Molecular Genetics and Genomics 293, no. 1: 237–248. 10.1007/s00438-017-1382-5. 29043489

[mec70360-bib-0082] Ortiz‐Alvarado, Y. , and B. Rivera‐Marchand . 2020. “Worker Queens? Behavioral Flexibility of Queens in the Little Fire Ant *Wasmannia auropunctata* .” Frontiers in Ecology and Evolution 8, no. August: 241. 10.3389/fevo.2020.00241.

[mec70360-bib-0083] Pantano, L. 2025. DEGreport. Bioconductor. http://bioconductor.org/packages/DEGreport/.

[mec70360-bib-0084] Peeters, C. 1997. “Morphologically “Primitive” Ants: Comparative Review of Social Characters, and the Importance of Queen–Worker Dimorphism.” In The Evolution of Social Behaviour in Insects and Arachnids, edited by B. J. Crespi and J. C. Choe . Cambridge University Press. 10.1017/CBO9780511721953.019.

[mec70360-bib-0085] Penick, C. A. , M. Ghaninia , K. L. Haight , et al. 2021. “Reversible Plasticity in Brain Size, Behaviour and Physiology Characterizes Caste Transitions in a Socially Flexible Ant ( *Harpegnathos saltator* ).” Proceedings of the Royal Society B: Biological Sciences 288, no. 1948: 20210141. 10.1098/rspb.2021.0141. PMC8059678 33849311

[mec70360-bib-0086] Petersen‐Braun, M. 1975. “Untersuchungen zur Sozialen Organisation der Pharaoameise Monomorium pharaonis (L.) (Hymenoptera, Formicidae) I. Der Brutzyklus und seine Steuerung durch Populationseigene Faktoren.” Insectes Sociaux 22, no. 3: 269–291. 10.1007/BF02223077.

[mec70360-bib-0087] Price, G. M. 1973. “Protein and Nucleic Acid Metabolism in Insect Fat Body.” Biological Reviews 48, no. 3: 333–372. 10.1111/j.1469-185X.1973.tb01006.x. 4201066

[mec70360-bib-0088] Qiu, B. , X. Dai , P. Li , et al. 2022. Canalized Gene Expression During Development Mediates Caste Differentiation in Ants. In Nature Ecology & Evolution. https://www.nature.com/articles/s41559‐022‐01884‐y. 10.1038/s41559-022-01884-y PMC9630140 36192540

[mec70360-bib-0089] Schindelin, J. , I. Arganda‐Carreras , E. Frise , et al. 2012. “Fiji: An Open‐Source Platform for Biological‐Image Analysis.” Nature Methods 9, no. 7: 676–682. 10.1038/nmeth.2019. 22743772 PMC3855844

[mec70360-bib-0090] Seeley, T. D. 1982. “Adaptive Significance of the Age Polyethism Schedule in Honeybee Colonies.” Behavioral Ecology and Sociobiology 11, no. 4: 287–293. 10.1007/BF00299306.

[mec70360-bib-0091] Smith, C. R. , A. L. Toth , A. V. Suarez , and G. E. Robinson . 2008. “Genetic and Genomic Analyses of the Division of Labour in Insect Societies.” Nature Reviews Genetics 9, no. 10: 735–748. 10.1038/nrg2429. 18802413

[mec70360-bib-0092] Soneson, C. , M. I. Love , and M. D. Robinson . 2016. Differential Analyses for RNA‐Seq: Transcript‐Level Estimates Improve Gene‐Level Inferences. F1000Research. 10.12688/f1000research.7563.1. PMC4712774 26925227

[mec70360-bib-0093] Szathmáry, E. , and J. M. Smith . 1995. “The Major Evolutionary Transitions.” Nature 374, no. 6519: 227–232. 10.1038/374227a0. 7885442

[mec70360-bib-0094] Taylor, B. A. , A. Cini , C. D. R. Wyatt , M. Reuter , and S. Sumner . 2021. “The Molecular Basis of Socially Mediated Phenotypic Plasticity in a Eusocial Paper Wasp.” Nature Communications 12, no. 1: 1. 10.1038/s41467-021-21095-6. PMC7859208 33536437

[mec70360-bib-0095] The UniProt Consortium . 2023. “UniProt: The Universal Protein Knowledgebase in 2023.” Nucleic Acids Research 51, no. D1: D523–D531. 10.1093/nar/gkac1052. 36408920 PMC9825514

[mec70360-bib-0096] Toth, A. L. , and G. E. Robinson . 2007. “Evo‐Devo and the Evolution of Social Behavior.” Trends in Genetics 23, no. 7: 334–341. 10.1016/j.tig.2007.05.001. 17509723

[mec70360-bib-0097] Traniello, J. F. A. 1982. “Population Structure and Social Organization in the Primitive Ant *Amblyopone pallipes* (*Hymenoptera: Formicidae*).” Psyche: A Journal of Entomology 89, no. 1–2: 1–2. 10.1155/1982/79349.

[mec70360-bib-0098] Treidel, L. A. , P. Goswami , and C. M. Williams . 2023. “Changes in Mitochondrial Function Parallel Life History Transitions Between Flight and Reproduction in Wing Polymorphic Field Crickets.” American Journal of Physiology. Regulatory, Integrative and Comparative Physiology 324, no. 6: R735–R746. 10.1152/ajpregu.00191.2022. 37036301

[mec70360-bib-0099] Tripet, F. , and P. Nonacs . 2004. “Foraging for Work and Age‐Based Polyethism: The Roles of Age and Previous Experience on Task Choice in Ants.” Ethology 110, no. 11: 863–877.

[mec70360-bib-0100] Vaser, R. , I. Sović , N. Nagarajan , and M. Šikić . 2017. “Fast and Accurate de Novo Genome Assembly From Long Uncorrected Reads.” Genome Research 27, no. 5: 737–746. 10.1101/gr.214270.116. 28100585 PMC5411768

[mec70360-bib-0101] Vieira, A. S. , W. D. Fernandes , and W. F. Antonialli‐Junior . 2012. “Behavioral Differentiation and Ovarian Development of Unmated Gynes, Queens, and Workers of *Ectatomma vizottoi* Almeida 1987 (Formicidae, Ectatomminae).” Psyche (Cambridge, Mass.) 2012, no. 1: 349896. 10.1155/2012/349896.

[mec70360-bib-0102] von Wyschetzki, K. , O. Rueppell , J. Oettler , and J. Heinze . 2015. “Transcriptomic Signatures Mirror the Lack of the Fecundity/Longevity Trade‐Off in Ant Queens.” Molecular Biology and Evolution 32, no. 12: 3173–3185. 10.1093/molbev/msv186. 26341296 PMC5009957

[mec70360-bib-0103] Wheeler, D. E. 1986. “Developmental and Physiological Determinants of Caste in Social Hymenoptera: Evolutionary Implications.” American Naturalist 128, no. 1: 13–34. 10.1086/284536.

[mec70360-bib-0104] Wheeler, W. M. 1900. “The Habits of Ponera and Stigmatomma.” Biological Bulletin 2, no. 2: 43–69. 10.2307/1535733.

[mec70360-bib-0105] Wheeler, W. M. 1911. “The Ant‐Colony as an Organism.” Journal of Morphology 22, no. 2: 307–325.

[mec70360-bib-0106] Wilson, E. O. 1953. “The Origin and Evolution of Polymorphism in Ants.” Quarterly Review of Biology 28, no. 2: 136–156. 10.1086/399512. 13074471

[mec70360-bib-0107] Wilson, E. O. 1976. “Behavioral Discretization and the Number of Castes in an Ant Species.” Behavioral Ecology and Sociobiology 1, no. 2: 141–154. 10.1007/BF00299195.

[mec70360-bib-0108] Wilson, E. O. 1978. “Division of Labor in Fire Ants Based on Physical Castes (Hymenoptera: Formicidae: Solenopsis).” Journal of the Kansas Entomological Society 4: 615–636.

[mec70360-bib-0109] Winston, M. L. 1991. The Biology of the Honey Bee. Harvard University Press.

[mec70360-bib-0110] Woodard, S. H. , G. Bloch , M. R. Band , and G. E. Robinson . 2013. “Social Regulation of Maternal Traits in Nest‐Founding Bumble Bee ( *Bombus terrestris* ) Queens.” Journal of Experimental Biology 216, no. 18: 3474–3482. 10.1242/jeb.087403. 23966589 PMC4074288

[mec70360-bib-0111] Xu, H. , and T. J. Colgan . 2025. “Localized Tissue‐Specific Gene Expression and Gene Duplications Are Important Sources of Social Morph Differences in a Social Bumblebee.” Molecular Biology and Evolution 42, no. 4: msaf063. 10.1093/molbev/msaf063. 40146539 PMC11968646

