# Supplementary material for: Unmated Queens Show Worker‐Like Behaviour and Gene Expression in Polygynous Colonies of the Ant Stigmatomma pallipes
Source: Mol Ecol. 2026 Apr 29;35:e70360. doi: 10.1111/mec.70360 (PMC13126620; doi:10.1111/mec.70360)
Supplement: Supplementary file 1 — Figure S1: Limited variation across observations in queen behaviour. The scatterplot displays the first two principal components from a principal component analysis (PCA) based on 13 scored behaviours for 35 queens across the five observations. The percentage of variance explained by each PC is provided in parentheses. Ellipses represent 95% confidence intervals around group centroids, colours indicate different observations, small dots correspond to individual queens, large dots to the group centroid. Figure S2: Mobility variation among Stigmatomma pallipes queens from monogynous and polygynous colonies allowed the selection of samples for further analyses. The dashed blue line at 4 mm indicates the threshold used to differentiate low mobility (LM) and high mobility (HM) polygynous queens. Indicated as green triangles are queens that were selected for mating status, ovarian development, behaviour, and gene expression in the brain and fat body. Colony identity is provided on the x‐axis, while queen mobility (estimated marginal mean for polygynous, mean for monogynous queens) is provided on the y‐axis. Figure S3: Clusters of differentially expressed genes (DEGs) that show similar expression differences across individual types for (A–D) the brain and (E) the fat body. Each cluster represented less than 5% of the number of DEGs. Each dot represents the z‐score‐transformed mean value of one gene, calculated from all individuals of that category. [file MEC-35-e70360-s002.docx]

**Unmated queens show worker-like behavior and gene expression in polygynous colonies of the ant *Stigmatomma pallipes***

**Maximilian F. Bolder^1^, Jannik S. Möllmann^1^, Thomas J. Colgan^1*^, Romain Libbrecht^1,2*^**

*contributed equally

^1^Institute of Organismic and Molecular Evolution, Johannes Gutenberg University, Mainz, Germany

^2^Insect Biology Research Institute, UMR 7261, CNRS, University of Tours, Tours, France

Correspondence: [maximilianbolder@gmail.com](mailto:maximilianbolder@gmail.com)

**Supplementary Figures S1-3**


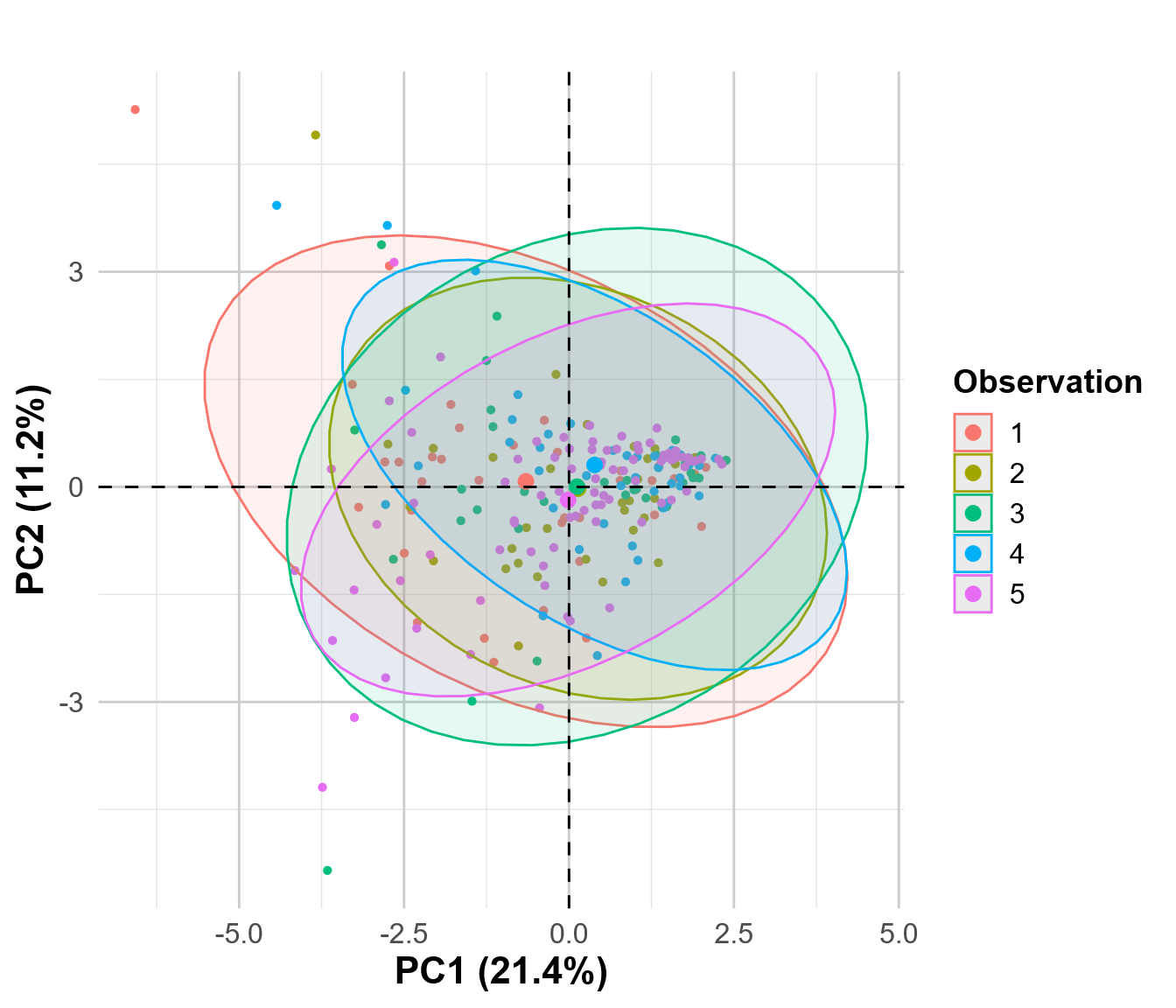


**Supplementary Figure S1: Limited variation across observations in queen behavior.** The scatterplot displays the first two principal components from a principal component analysis (PCA) based on 13 scored behaviors for 35 queens across the five observations. The percentage of variance explained by each PC is provided in parentheses. Ellipses represent 95% confidence intervals around group centroids, colors indicate different observations, small dots correspond an individual queen, big dots the group centroid.


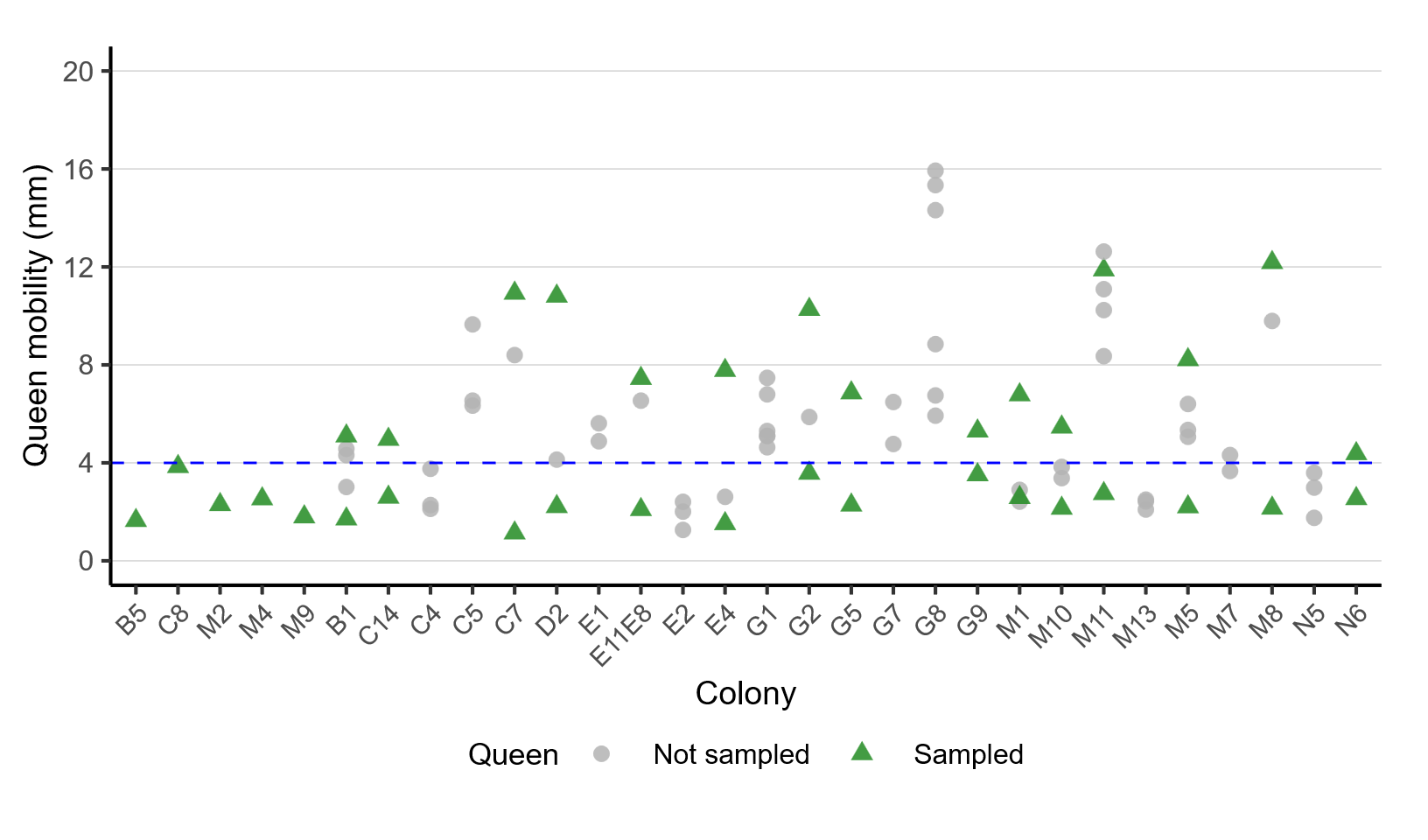
**Supplementary Figure S2:** **Mobility variation among *Stigmatomma pallipes* queens from monogynous and polygynous colonies allowed the selection of samples for further analyses.** The dashed blue line at 4 mm indicates the threshold used to differentiate low mobility (LM) and high mobility (HM) polygynous queens. Indicated as green triangles are queens that were selected for mating status, ovarian development, behavior, and gene expression in the brain and fat body. Colony identity is provided on the x-axis while queen mobility (estimated marginal mean for polygynous, mean for monogynous queens) is provided on the y-axis.


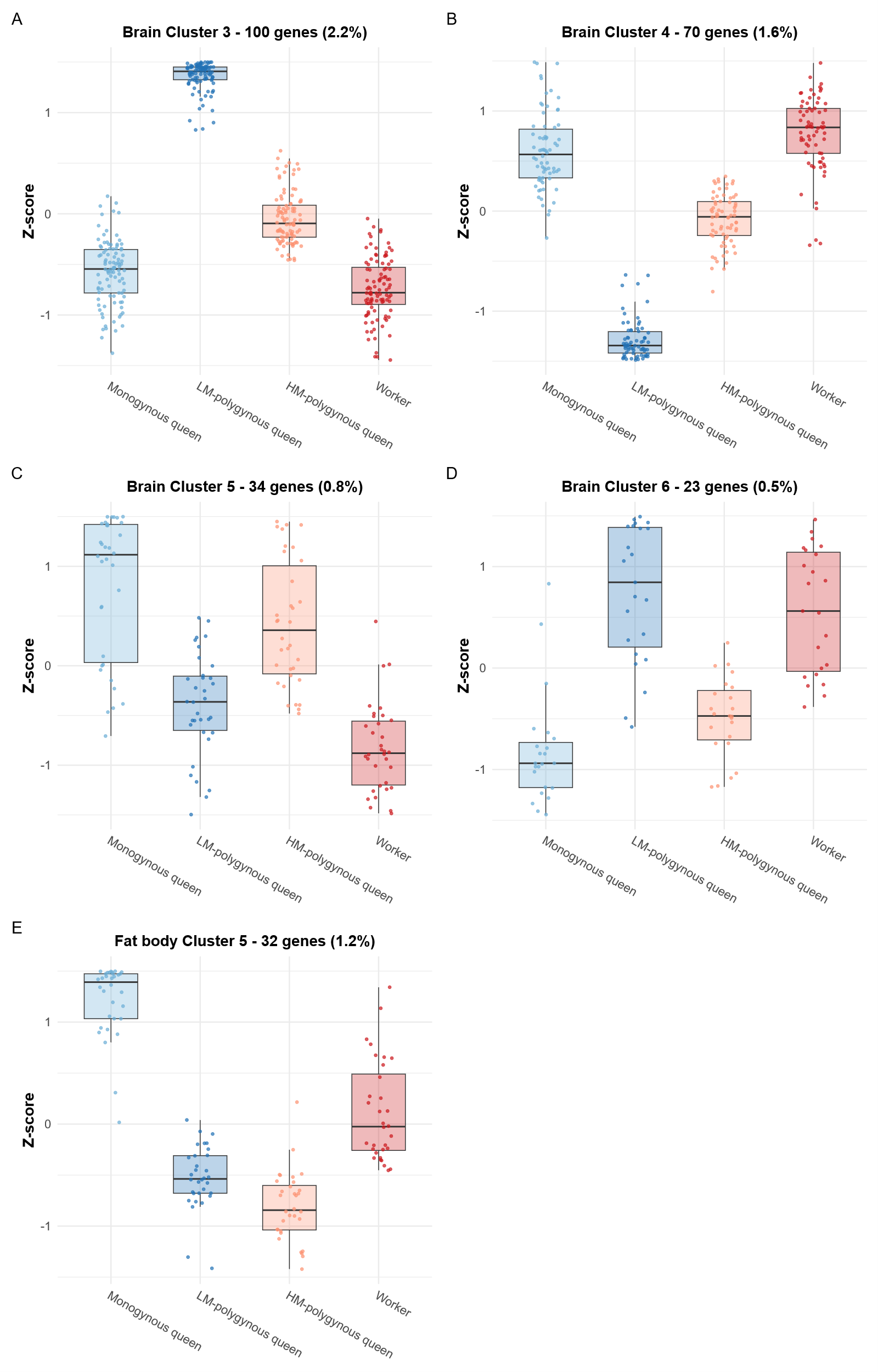


**Supplementary Figure 3: Clusters of differentially expressed genes (DEGs) that show similar expression differences across individual types for A-D) the brain and E) the fat body.** Each cluster represented less than 5% of the number of DEGs. Each dot represents the z-score-transformed mean value of one gene, calculated from all individuals of that category.
